# Data-driven identification of post-acute SARS-CoV-2 infection subphenotypes

**DOI:** 10.1038/s41591-022-02116-3

**Published:** 2022-12-01

**Authors:** Hao Zhang, Chengxi Zang, Zhenxing Xu, Yongkang Zhang, Jie Xu, Jiang Bian, Dmitry Morozyuk, Dhruv Khullar, Yiye Zhang, Anna S. Nordvig, Edward J. Schenck, Elizabeth A. Shenkman, Russell L. Rothman, Jason P. Block, Kristin Lyman, Mark G. Weiner, Thomas W. Carton, Fei Wang, Rainu Kaushal

**Affiliations:** 1 Department of Population Health Sciences, Weill Cornell Medicine, New York, NY USA; 2 Department of Health Outcomes Biomedical Informatics, University of Florida, Gainesville, FL USA; 3 Department of Neurology, Weill Cornell Medicine, New York, NY USA; 4 Department of Medicine, Division of Pulmonary and Critical Care Medicine, Weill Cornell Medicine, New York, NY USA; 5 Center for Health Services Research, Vanderbilt University Medical Center, Nashville, TN USA; 6 Department of Population Medicine, Harvard Pilgrim Health Care Institute, Harvard Medical School, Boston, MA USA; 7 Louisiana Public Health Institute, New Orleans, LA USA

**Keywords:** Diseases, Health care

## Abstract

The post-acute sequelae of SARS-CoV-2 infection (PASC) refers to a broad spectrum of symptoms and signs that are persistent, exacerbated or newly incident in the period after acute SARS-CoV-2 infection. Most studies have examined these conditions individually without providing evidence on co-occurring conditions. In this study, we leveraged the electronic health record data of two large cohorts, INSIGHT and OneFlorida+, from the national Patient-Centered Clinical Research Network. We created a development cohort from INSIGHT and a validation cohort from OneFlorida+ including 20,881 and 13,724 patients, respectively, who were SARS-CoV-2 infected, and we investigated their newly incident diagnoses 30–180 days after a documented SARS-CoV-2 infection. Through machine learning analysis of over 137 symptoms and conditions, we identified four reproducible PASC subphenotypes, dominated by cardiac and renal (including 33.75% and 25.43% of the patients in the development and validation cohorts); respiratory, sleep and anxiety (32.75% and 38.48%); musculoskeletal and nervous system (23.37% and 23.35%); and digestive and respiratory system (10.14% and 12.74%) sequelae. These subphenotypes were associated with distinct patient demographics, underlying conditions before SARS-CoV-2 infection and acute infection phase severity. Our study provides insights into the heterogeneity of PASC and may inform stratified decision-making in the management of PASC conditions.

## Abstract (web summary)

Machine learning applied to electronic health records in two US cohorts from the RECOVER initiative identified four Long-COVID subphenotypes that differ in the involvement of organ systems, previous SARS-CoV-2 infection severity and underlying conditions.

## Main

The ongoing global pandemic of Coronavirus Disease 2019 (COVID-19) caused by severe acute respiratory syndrome coronavirus 2 (SARS-CoV-2) infection has impacted hundreds of millions of people’s lives. Existing studies have provided evidence that many symptoms and signs could be persistent, exacerbated or newly present after the acute phase of SARS-CoV-2 infection, referred to as post-acute sequelae of SARS-CoV-2 infection (PASC)^1,2^, which involve multiple organ systems, including cardiovascular^3^, mental^4^, metabolic^5^, renal^6^ and others. There have been various ongoing efforts into investigating the underlying biological mechanisms of PASC^7–9^, which have typically been conducted in small patient cohorts. Large-scale clinical observational cohort studies can provide useful insights into PASC that may help develop effective mechanistic hypotheses and inform targeted treatments. Most existing observational studies investigated the PASC conditions individually (for example, by examining the incidence^10^, excess burden^11^ or prevalence^12^ of each symptom or condition in the post-acute period for patients infected with SARS-CoV-2 relative to non-infected individuals). Their co-appearance patterns (or subphenotypes)—that is, to what extent PASC symptoms and conditions co-appear or develop disproportionately in certain patient populations—can potentially help reveal the pathophysiology behind PASC by disentangling their phenotypical heterogeneities. Existing studies on this topic have been limited, with one study that identified PASC subphenotypes from the reported symptoms of a cohort including 233 patients with COVID-19 (ref. ^13^). More comprehensive analysis involving broader sets of PASC conditions with larger patient cohorts is needed.

We developed a machine learning approach to derive PASC subphenotypes based on newly incident conditions in the post-acute SARS-CoV-2 infection period (defined as 30–180 days after the confirmed infection) of patients with COVID-19. We leveraged the electronic health record (EHR) repositories of two large clinical research networks (CRNs) from the national Patient-Centered Clinical Research Network (PCORnet): the INSIGHT network^14^, which includes 12 million patients in the New York City (NYC) area, and the OneFlorida+ network^15^, which includes 19 million patients from Florida, Georgia and Alabama. We examined the incidence of 137 diagnosis categories derived from the Clinical Classifications Software Refined (CCSR) categories^16^ that were potentially related to PASC, and we leveraged a topic modeling (TM) approach^17^ to learn the co-incidence patterns of these diagnosis conditions, based on which the PASC subphenotypes were derived by clustering.

## Results

### Overall pipeline

**Fig. 1:**
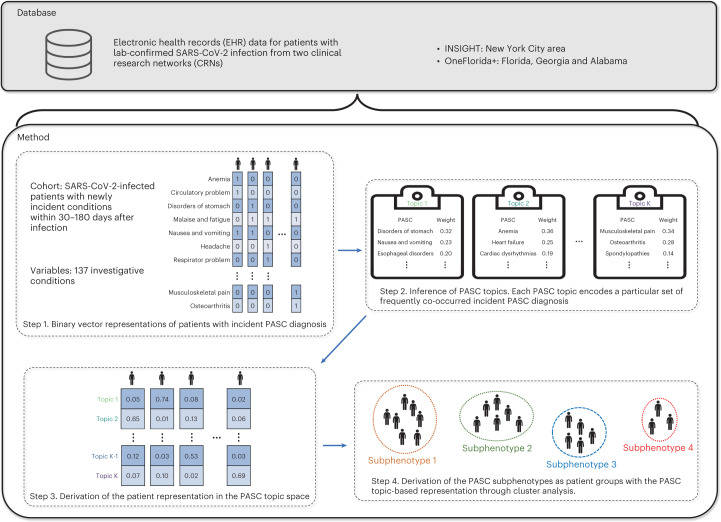
Data curation and the subphenotyping pipeline. To study the subphenotypes for patients with PASC, we constructed cohorts of SARS-CoV2-positive and SARS-CoV2-negative individuals from the INSIGHT and OneFlorida+ CRNs. After obtaining high-dimensional binary representations of patients with PASC diagnoses (step 1), our algorithm learned PASC topics (step 2) and inferred the patient representations in the low-dimensional PASC topic space (step 3) through a TM approach. Finally, PASC subphenotypes were derived as patient clusters based on representations by PASC topics (step 4).

Figure 1 shows our overall analytics pipeline. Using the two CRNs, we extracted the EHR of patients who had positive nucleic acid amplification or antigen viral tests for SARS-CoV-2 from March 2020 to November 2021. A list of 137 potential PASC diagnosis categories (Methods) was compiled, and only patients who have had documented new incidences of these conditions in the post-acute infection period were retained. Each patient was initially characterized as a 137-dimensional binary vector encoding whether a particular condition appeared in his/her post-acute infection period or not (step 1). Next, a set of ‘PASC topics’ was learned from these vectors; PASC topics in this context refer to a group of conditions that present together based on their incident probabilities (step 2). Then, the patients are further characterized by these topics according to how much each topic was represented in their post-acute period records (step 3). Finally, a clustering procedure was performed from topic-induced characterizations to identify the subphenotypes (step 4).

### Study cohorts

Our study included 20,881 and 13,724 patients from the INSIGHT and OneFlorida+ CRNs who were virally tested positive for SARS-CoV-2, respectively (Methods). The diagnosis information in International Classification of Diseases (ICD)-10 codes of these patients in the post-SARS-CoV-2 infection period was analyzed. The INSIGHT cohort had a median age of 58.0 years (interquartile range (IQR) (42.0–70.0)) and a median Area Deprivation Index (ADI)^18^ of 15.0 (IQR (6.0–25.0)). It consisted of 58.37% female (*n* = 12,188), 33.59% White (*n* = 7,013) and 22.85% Black (*n* = 4,771). The OneFlorida+ cohort included patients with a median age of 51.0 years (IQR (35.0–65.0)) and is more socioeconomically deprived, with a median ADI of 59.0 (IQR (42.0–76.0)). It had a greater proportion of White patients (52.28%, *n* = 7,175).

Within the INSIGHT cohort, 33.04% of the patients had a confirmed SARS-CoV-2 infection from March to June 2020 (compared to 8.83% from the OneFlorida+ cohort). This reflected the centrality of NYC during the first wave of COVID-19 in the United States. More patients from OneFlorida+ were tested positive from July to October 2020 (26% versus 6% for INSIGHT). The summary statistics of the two cohorts are available in Table 1 (with more details provided in Supplementary Table 1).

**Table 1:** Characteristics of the INSIGHT cohort (for development) and the OneFlorida+ cohort (for validation)

Characteristics	INSIGHT cohort	OneFlorida+ cohort
	Development	Validation
No. of patients	20,881	13,724
Age in years, median (IQR)^a^	58.0 (42.0–70.0)	51.0 (35.0–65.0)
Age group, no. (%)		
20 to <40 years	4,603 (22.04%)	4,284 (31.22%)
40 to <55 years	4,522 (21.66%)	3,318 (24.18%)
55 to <65 years	4,308 (20.63%)	2,528 (18.42%)
65 to <75 years	3,810 (18.25%)	1,923 (14.01%)
75 to <85 years	2,553 (12.22%)	1,178 (8.58%)
85+ years	1,085 (5.20%)	493 (3.59%)
Sex, no. (%)		
Female	12,188 (58.37%)	8,468 (61.70%)
Male	8,692 (41.63%)	5,255 (38.29%)
Other/Missing	1 (0.00%)	1 (0.00%)
Race, no. (%)		
Asian	945 (4.52%)	144 (1.05%)
Black or African American	4,771 (22.85%)	4,076 (29.70%)
White	7,013 (33.59%)	7,175 (52.28%)
Other	6,238 (29.87%)	2,123 (15.47%)
Missing	1,914 (9.17%)	206 (1.50%)
Ethnic group, no. (%)		
Hispanic: Yes	6,838 (32.74%)	2,881 (20.99%)
Hispanic: No	12,248 (58.66%)	9,329 (67.98%)
Hispanic: Other/Missing	1,795 (8.60%)	1,514 (11.03%)
ADI, median (IQR)	15.0 (6.0–25.0)	59.0 (42.0–76.0)
Healthcare utilization^b^ in the past 3 years, no. (%)		
Inpatient = 0	14,631 (70.07%)	7,437 (54.19%)
0 < Inpatient < 5	5,433 (26.03%)	4,213 (30.70%)
Inpatient ≥ 5	817 (3.91%)	2,074 (15.11%)
Outpatient = 0	1,241 (5.94%)	2,759 (20.10%)
0 < Outpatient < 5	3,607 (17.28%)	2,556 (18.63%)
Outpatient ≥ 5	16,033 (76.78%)	8,409 (61.27%)
Emergency = 0	11,321 (54.22%)	4,908 (35.76%)
0 < Emergency < 5	7,705 (36.90%)	4,851 (35.35%)
Emergency ≥ 5	1,855 (8.89%)	3,965 (28.89%)
Index time period of patient, no. (%)		
3/20–6/20	6,899 (33.04%)	1,212 (8.83%)
7/20–10/20	1,180 (5.65%)	3,570 (26.01%)
11/20–2/21	8,714 (41.73%)	3,988 (29.06%)
3/21–6/21	3,390 (16.24%)	1,531 (11.16%)
7/21–11/21	698 (3.34%)	3,423 (24.94%)
Acute phase severities of COVID-19 (−1 to ~16 days)^c^, no. (%)		
Hospitalized	9,076 (43.47%)	5,036 (36.69%)
Ventilation	495 (2.37%)	465 (3.39%)
Critical care	1,144 (5.48%)	833 (6.07%)

^a^IQR, interquartile range.

^b^For the healthcare utilization in the past 3 years, including inpatient, outpatient and emergency visits, we binned the number of visits into five levels.

^c^Time range for acute phase severities is from 1 day before index date to 16 days after index date.

### Potential PASC co-incidence patterns

We compiled a list of 137 potentially PASC-related diagnosis groups defined by ICD-10 diagnosis codes and CCSR categories^16^ (Supplementary Table 2). We first investigated the co-incidence patterns across different diagnoses 30–180 days after confirmed SARS-CoV-2 infection through probabilistic TM^19^, which was originally proposed for learning word co-occurrence patterns in documents with different semantic topics. With this approach (Methods), we identified ten distinct ‘PASC topics’, each of which is characterized by a unique post-acute infection incidence probability distribution across the 137 individual conditions.

**Fig. 2:**
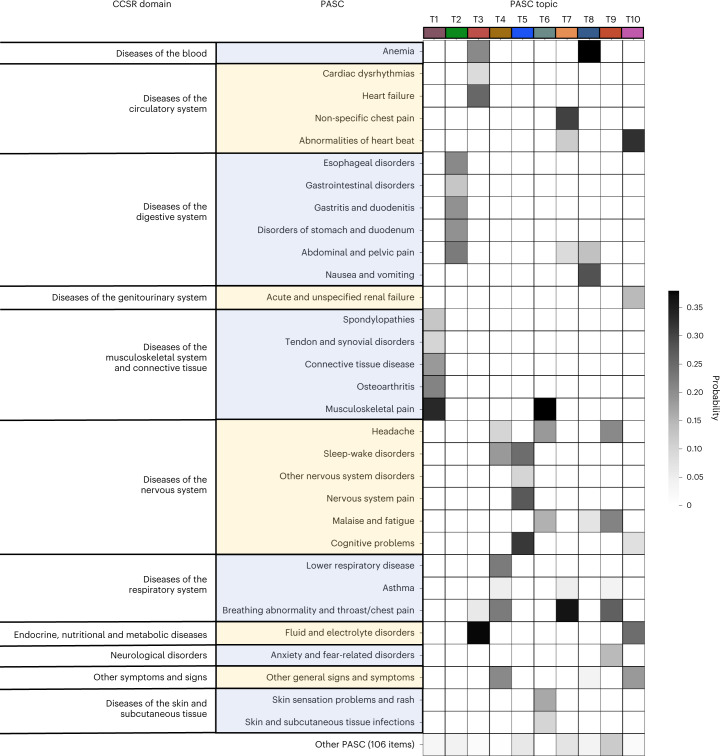
Heat map of PASC topics learned from the INSIGHT cohort. Each row denotes a potential PASC diagnosis category defined by ICD-10 codes classified through CCSR, and each column denotes a particular PASC topic. Each PASC topic is characterized by a unique post-acute incidence probability distribution over all 137 individual potential PASC diagnosis categories. Source data

Figure 2 shows the heat map matrix of topics learned from the INSIGHT cohort. Each column is a learned topic, and each row is a potential PASC condition category (we demonstrated 31 in the heat map and aggregated the remaining 106 because none of their incident probabilities exceeded 0.1 in any of the learned topics). Each entry in the matrix corresponds to the incident probability of the specific PASC condition in the corresponding topic. Topics T1, T2 and T5 were concentrated on the conditions of the musculoskeletal, digestive and nervous systems, respectively. Topics T4, T7 and T9 included respiratory conditions mixed with sleep disorder and anxiety along with symptoms such as headache and chest pain. Topic T3 included fluid and electrolyte disorders combined with anemia and cardiac complications. Topic T6 was dominated by musculoskeletal and skin conditions as well as headache and fatigue. Topic T8 mainly comprised anemia and digestive system conditions. Topic T10 contained a mix of circulatory conditions, renal failure, fluid and electrolyte disorders and others.

### Potential PASC subphenotypes

**Fig. 3:**
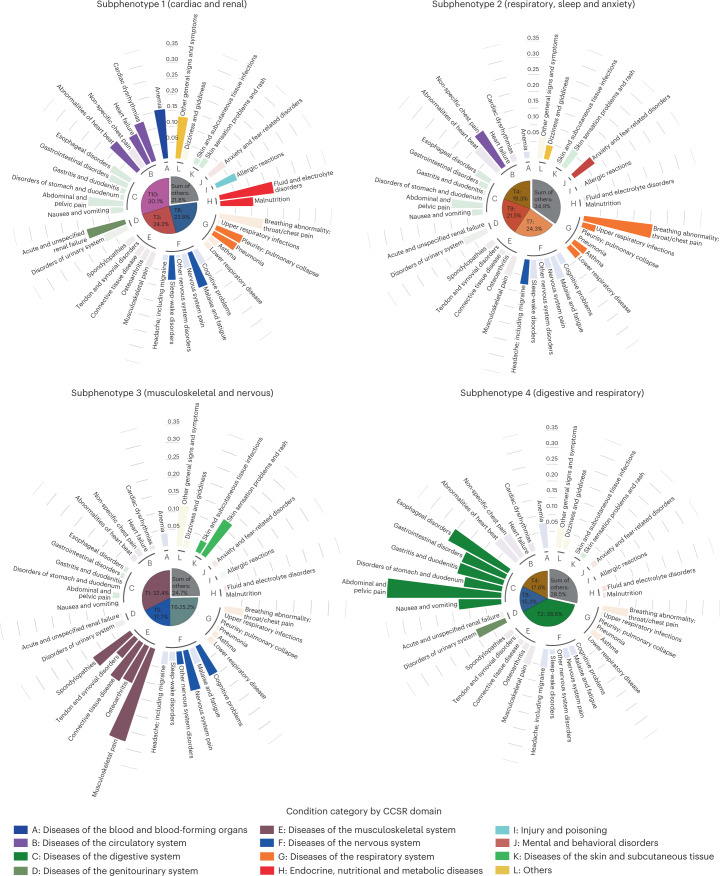
Incidence rates of potential PASC conditions in each subphenotype for the INSIGHT cohort (more numerical results are in Supplementary Table 4), where potential PASC conditions were grouped into different categories shown in different colored bars outside the center pie chart. A condition was highlighted from a particular subphenotype where it had the highest incidence rate compared to other subphenotypes. The center pie chart of each subphenotype showed the mean topic proportions (we showed the three most dominant topics and aggregated the rest) of the patients belonging to each subphenotype, and the colors and definitions of the topics were consistent with Fig. 2. Larger percentage of a particular topic shows more representations of this topic within this subphenotype. Source data

**Extended Data Fig. 1:**
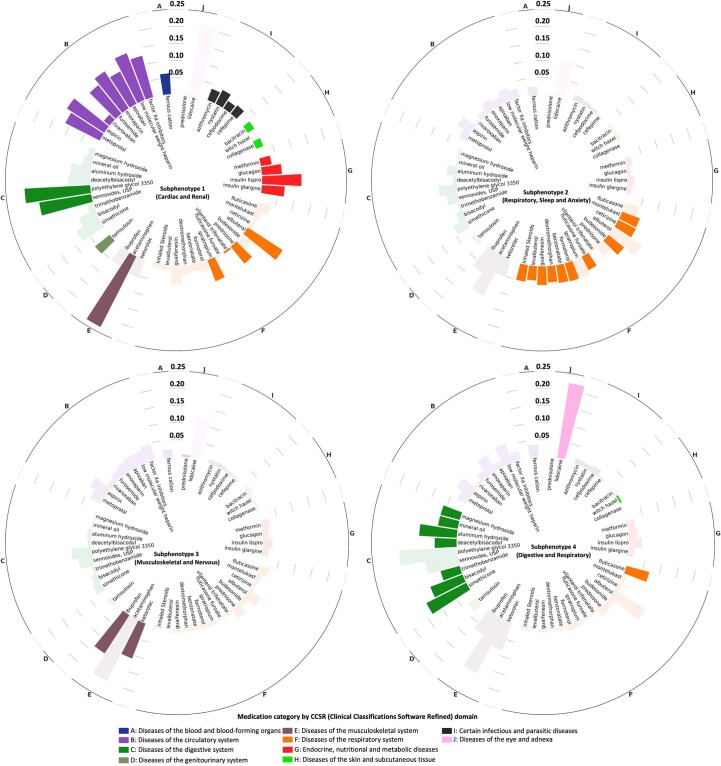
Circos plot for the INSIGHT subphenotypes with newly incident medications. The prevalence of incident prescriptions of medications in the post-acute infection period for each subphenotype on the INSIGHT cohort, where medications are grouped into different categories shown by different colors. The most prevalent medication in each subphenotype is highlighted. Source data

After identifying potential PASC topics, we could describe patients with PASC using these topics and derive potential PASC subphenotypes as patient clusters (Methods). Specifically, four subphenotypes were identified from the INSIGHT cohort. Table 2 summarizes their characteristics with respect to patient demographics, medical utilizations and pre-existing conditions in the baseline period and disease severity in the acute phase according to medical utilization (more details provided in Supplementary Table 3). Across different subphenotypes, we demonstrated the incidence rates of specific potential PASC conditions (Fig. 3, with numerical results in Supplementary Table 4) and medication prescriptions (Extended Data Fig. 1). We characterized these subphenotypes in detail as follows.

**Table 2:** Characteristics of the identified subphenotypes on the INSIGHT cohort

Variable	Total	Subphenotype 1 (cardiac and renal)	Subphenotype 2 (respiratory, sleep and anxiety)	Subphenotype 3 (musculoskeletal and nervous)	Subphenotype 4 (digestive and respiratory)	*P* value^a^	Post hoc analysis^a^
No. of patients (%)	20,881 (100%)	7,047 (33.75%)	6,838 (32.75%)	4,879 (23.37%)	2,117 (10.14%)		
Age in years, median (IQR^b^)	58.0 (42.0–70.0)	65.0 (52.0–75.0)	51.0 (35.0–64.0)	57.0 (42.0–69.0)	54.0 (39.0–67.0)	0	2 vs. 1, 3 vs. 1, 4 vs. 1, 3 vs. 2, 4 vs. 2, 4 vs. 3
Female, no. (%)	12,188 (58.37%)	3,627 (51.47%)	4,294 (62.80%)	2,962 (60.71%)	1,305 (61.64%)	4.91E × 46	1 vs. 2, 1 vs. 3, 1 vs. 4, 2 vs. 3
Race, no. (%)							
Asian	945 (4.52%)	278 (3.94%)	339 (4.96%)	237 (4.86%)	91 (4.3%)	0.019	1 vs. 2, 1 vs. 3
Black or African American	4,771 (22.85%)	1,752 (24.86%)	1,482 (21.67%)	1,116 (22.87%)	421 (19.89%)	4.99E × 7	1 vs. 2, 1 vs. 3, 1 vs. 4, 3 vs. 4
White	7,013 (33.59%)	2,407 (34.16%)	2,255 (32.98%)	1,670 (34.23%)	681 (32.17%)	0.174	–
Other	6,238 (29.87%)	2,052 (29.12%)	2,041 (29.85%)	1,411 (28.92%)	734 (34.67%)	5.13E × 6	1 vs. 4, 2 vs. 4, 3 vs. 4
Missing	1,914 (9.17%)	558 (7.92%)	721 (10.54%)	445 (9.12%)	190 (8.97%)	2.43E × 6	1 vs. 2, 1 vs. 3, 2 vs. 3, 2 vs. 4
Hispanic, no. (%)	6,838 (32.74%)	2,227 (31.60%)	2,277 (33.30%)	1,554 (31.85%)	780 (36.84%)	3.92E × 5	1 vs. 2, 1 vs. 4, 2 vs. 4, 3 vs. 4
ADI, median (IQR)	15.0 (6.0–25.0)	16.0 (7.0–25.0)	14.0 (6.0–24.0)	15.0 (6.0–23.5)	15.0 (7.0–25.0)	4.21E × 9	2 vs. 1
Index time period of patient, no. (%)							
3/20–6/20	6,899 (33.04%)	2,634 (37.38%)	2,040 (29.83%)	1,554 (31.85%)	671 (31.7%)	8.52E × 21	1 vs. 2, 1 vs. 3, 1 vs. 4, 2 vs. 3
7/20–10/20	1,180 (5.65%)	394 (5.59%)	390 (5.70%)	245 (5.02%)	151 (7.13%)	6.06E × 3	1 vs. 4, 2 vs. 4, 3 vs. 4
11/20–2/21	8,714 (41.73%)	2,708 (38.43%)	3,016 (44.11%)	2,129 (43.64%)	861 (40.67%)	4.75 E× 12	1 vs. 2, 1 vs. 3, 2 vs. 4, 3 vs. 4
3/21–6/21	3,390 (16.24%)	1,112 (15.78%)	1,113 (16.28%)	794 (16.27%)	371 (17.52%)	0.298	–
7/21–11/21	698 (3.34%)	199 (2.82%)	279 (4.08%)	157 (3.22%)	63 (2.98%)	3.47E × 4	1 vs. 2, 2 vs. 3, 2 vs. 4
Acute phase severities of COVID-19 (−1 to ~16 days), no. (%)							
Hospitalized	9,076 (43.47%)	4,309 (61.15%)	2,207 (32.28%)	1,855 (38.02%)	705 (33.3%)	1.05E × 301	1 vs. 2, 1 vs. 3, 1 vs. 4, 2 vs. 3, 3 vs. 4
Ventilation	495 (2.37%)	339 (4.81%)	85 (1.24%)	54 (1.11%)	17 (0.8%)	2.58E × 59	1 vs. 2, 1 vs. 3, 1 vs. 4
Critical care	1144 (5.48%)	701 (9.95%)	210 (3.07%)	174 (3.57%)	59 (2.79%)	4.62E × 89	1 vs. 2, 1 vs. 3, 1 vs. 4

^a^*P* value with post hoc analysis: continuous variables were tested by one-way ANOVA, with Tukeyʼs honestly significant difference post hoc test) for normal distribution or by Kruskal–Wallis test (with Dunn post hoc test) for non-normal distribution; categorical variables were tested by one-sided Fisher’s exact test with pairwise Fisherʼs test for post hoc analysis. Post hoc analysis is used to uncover specific differences among subphenotypes—for example, 1 vs. 2: subphenotype 1 and 2 has significant difference.

^b^IQR, interquartile range.

Subphenotype 1 (cardiac and renal) consisted of 7,047 (33.75%) patients. It was dominated by cardiac-related, renal-related and circulation-related topics (T3, T8 and T10), including cardiac and circulatory conditions, renal failure, anemia and fluid and electrolyte disorders. Compared to other subphenotypes, patients in this subphenotype were older (median age, 65.0 years, IQR (52.0–75.0)) and had the highest proportion of males (48.53%). The patients also had a higher acute severity of COVID-19 (with the highest rate of hospitalization (61.15%), use of mechanical ventilation (4.81%) and critical care services (9.95%)). This subphenotype had the highest proportion of patients (37.38%) infected with SARS-CoV-2 during the first wave of the pandemic (March–June 2020). Patients in this subphenotype had a higher burden of pre-existing conditions than other subphenotypes, especially for blood, circulation and endocrine comorbidities, as well as a high level of incident prescriptions of medications for treating circulatory and endocrine conditions and anemia.

Subphenotype 2 (respiratory, sleep and anxiety) included 6,838 (32.75%) patients. It was dominated by respiratory conditions (topics T4, T7 and T9), sleep disorders, anxiety and symptoms such as headache and chest pain. This subphenotype had patients with a median age of 51.0 years (IQR (35.0–64.0)), female representation of 62.8% and hospitalization rate of 31.28% during the acute SARS-CoV-2 infection phase. It had the largest proportion of patients who tested positive for SARS-CoV-2 from November 2020 to November 2021 (64.47%). Patients in this subphenotype had higher baseline comorbidity burdens for respiratory conditions, such as upper respiratory sequelae and chronic obstructive pulmonary disease. These patients were associated with higher incident prescription rates for anti-asthma, anti-allergy and anti-inflammatory medications, including inhaled steroids, levalbuterol and montelukast.

Subphenotype 3 (musculoskeletal and nervous) consisted of 4,879 (23.37%) patients who mainly had musculoskeletal and nervous system sequelae (topics T1, T5 and T6), such as musculoskeletal pain, headaches and sleep-wake disorders. This subphenotype included patients with a median age of 57.0 years (IQR (42.0–69.0)), and 60.71% were female. It had the highest proportion of patients with more than five outpatient visits before SARS-CoV-2 infection (78.4%) and higher baseline comorbidity burdens of autoimmune and allergy conditions, such as rheumatoid arthritis and asthma, as well as other musculoskeletal and nervous system conditions, including soft tissue, bone and sleep disorders. This subphenotype was associated with more incident prescriptions for pain medications (for example, ibuprofen and ketorolac).

Subphenotype 4 (digestive and respiratory) included 2,117 (10.14%) patients mainly with digestive system and respiratory conditions (topics T2, T4 and T8). Patients in this subphenotype had a median age of 54.0 years (IQR (39.0–67.0)), 61.64% female, the highest rates of zero baseline emergency visits (57.06%) as well as the lowest rates of mechanical ventilation (0.8%) and critical care admission (2.79%) in the acute phase of SARS-CoV-2 infection. Compared to other subphenotypes, Subphenotype 4 had a lower overall burden of underlying conditions but a slightly higher prevalence of digestive system conditions, such as hematemesis, stomach and duodenum disorders and digestive system neoplasm, and more incident prescription digestive system medications.

For each subphenotype, we also provide the sex-disaggregated analysis results with respect to patient demographics, baseline and acute infection phase medical utilizations, prior condition prevalence and newly PASC incidence rates in Supplementary Table 5, which can help trigger effective mechanistic hypotheses of the different PASC subphenotypes.

### Comparison with patients without SARS-CoV-2 infection

**Extended Data Fig. 2:**
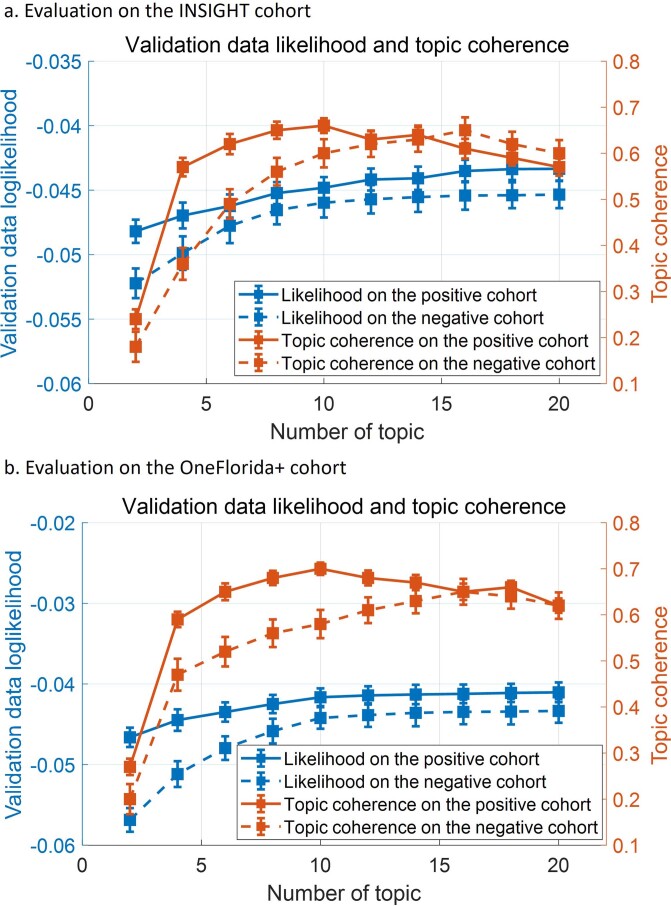
PASC topic curve for the INSIGHT and OneFlorida+ cohorts. Data likelihood and topic coherence with respect to different number of topics on a) the INSIGHT cohort and b) the OneFlorida+ cohort. For both criteria, larger values indicate better quality of the learned topics. We evaluated them on the group of patients who tested positive for SARS-CoV-2 infection (solid curves), and the group of patients who tested negative for SARS-CoV-2 infection (dashed curves). For each evaluation, we randomly split the corresponding patient group into 70% for training and the rest 30% for evaluation, and repeated this process for 100 times. The average evaluation results are shown as centers in the curves and standard deviations are shown as error bars. Source data

**Extended Data Fig. 3:**
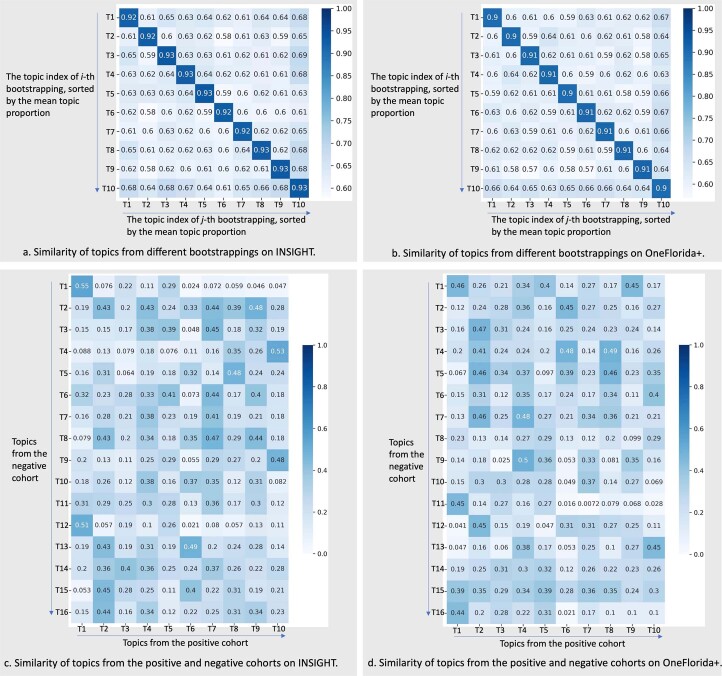
Topic robustness test for the INSIGHT and OneFlorida+ cohorts. The similarity of topics from different bootstrapping on a) the INSIGHT cohort and b) the OneFlorida+ cohort, which were used to evaluate the robustness of topics. c and d: the similarity of topics from the patients who tested positive and negative for SARS-CoV-2 infection on INSIGHT and OneFlorida+ cohorts, which were used to evaluate the consistency of topics learned from these two cohorts. Source data

To investigate how potential PASC co-incidence patterns in patients with confirmed SARS-CoV-2 infection differed from those without infection, we constructed a cohort of patients who visited the hospitals but were tested negative for SARS-CoV-2 with a rigorous matching process considering patient demographics and baseline characteristics. Extended Data Fig. 2a shows quantitatively how well the data can be modeled with respect to different numbers of topics for patients who tested positive versus negative for SARS-CoV-2 infection within the INSIGHT cohort. We observed that a larger number of topics was needed for characterizing patients who tested negative for SARS-CoV-2. In addition, we calculated the similarities between the topics learned from the patients who tested positive versus negative for SARS-CoV-2 infection and showed them as heat maps in Extended Data Fig. 3c, from which we could observe that the topics learned from COVID-19-positive and COVID-19-negative patients were not similar to each other. To further examine the concentration patterns of the learned topics on individual conditions, we calculated the entropy of each topic vector, such that higher entropy value indicated less concentration patterns—that is, the probability values comprising each topic vector were more evenly distributed across individual conditions. Supplementary Table 6 demonstrates the detailed entropy values of each topic learned from patients who tested positive for SARS-CoV-2 infection or not, from which we could observe that the topics learned from patients who tested negative displayed higher entropy values compared to patients who tested positive. These investigations suggest that the condition incidence patterns in the follow-up period for matched patients who tested negative were much less clear—that is, these conditions appeared more randomly without clear co-incidence patterns. This further strengthened the validity of our identified potential PASC subphenotypes as they were unique to patients who tested positive for SARS-CoV-2 infection.

**Fig. 4:**
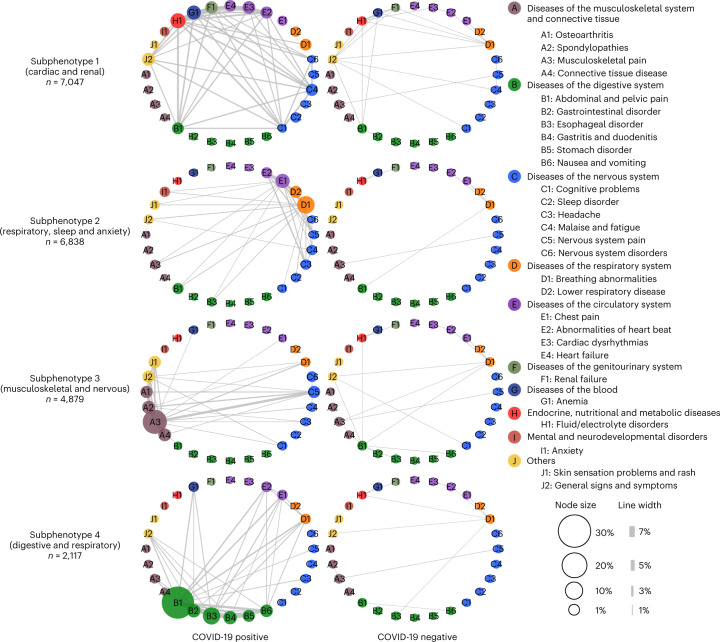
Differences in incidence patterns of selected PASC conditions (grouped by CCSR domains) in 30–180 days after COVID-19 lab test between positive and matched negative patients on the INSIGHT cohort. The bubbles in each network correspond to a PASC condition with their sizes proportional to the incidences in the particular subphenotype or matched controls. The edge linking a pair of bubbles indicates co-incidence of the corresponding potential PASC conditions, with its thickness proportional to the co-incidence rate, where lines are visible if the rate is larger than 1%. Source data

We also plotted heat maps of the post-acute co-incidence rate matrix of the 137 potential PASC conditions for patients in each subphenotype and their matched controls in Supplementary Fig. 1. We observed that patients in these PASC subphenotypes are associated with higher co-incidence rates of PASC conditions compared to their matched controls. We further visualized in Fig. 4 the network patterns of 28 selected potential PASC conditions whose incidence rates were larger than 1% in any of the PASC subphenotypes, where the nodes in each network are particular potential PASC conditions with their sizes proportional to the incidence rate in the records of patients from the corresponding group. Each line linking a pair of nodes indicates a co-incidence of that pair of potential PASC conditions, with its thickness proportional to the co-incidence rate in the corresponding group. Figure 4 shows that the conditions used to characterize each PASC subphenotype were clearly associated with larger-sized nodes, representing higher incidence rates. There were no clear differences in node sizes for the groups with matched controls. We also observed denser connections in PASC subphenotypes, which suggested that the potential PASC conditions did not appear independently, but rather collectively, and those larger nodes included more interconnected network hubs.

### Sensitivity analysis

Our analysis so far was based on a comprehensive list of 137 potential PASC conditions compiled from existing literature and clinician input. With respect to a specific patient cohort, it was challenging to guarantee that all of these conditions would be associated with excessive incidence risk in the follow-up period for patients who tested positive versus negative for SARS-CoV-2 infection due to population heterogeneity and incomplete information capture in the EHR. One key characteristic for TM was that it could effectively suppress the impact of conditions with low incidence rates in the EHR and drew more focus on the prevalent conditions (Fig. 2). However, it was still unclear if the subphenotypes would change if only the conditions that were associated with statistically significant excessive risks in the follow-up period for patients who tested positive for SARS-CoV-2 infection compared to patients who tested negative were considered.

We examined the robustness of the identified PASC subphenotypes in a more restricted set of PASC conditions. Specifically, with a high-dimensional propensity score (PS) adjustment pipeline^20,21^ and existing research into PASC^10,21,22^, we identified 44 PASC conditions (Supplementary Table 7) in the INSIGHT cohort that demonstrated statistically significant higher risk in the follow-up period for patients who tested positive versus negative for SARS-CoV-2 infection. Then, we implemented the same subphenotyping process based on these 44 PASC conditions. Finally, we quantified the overlap between these newly derived subphenotypes with the subphenotypes shown in Fig. 3.

Supplementary Fig. 2a demonstrates the learned PASC topics from this restricted condition set, where the optimal number of topics is determined according to Supplementary Fig. 2b. We further quantitatively compared these topics and the original set of topics learned from the 137 PASC conditions shown in Fig. 2 with cosine similarity, which is shown in Supplementary Fig. 2e as a heat map, suggesting that they are highly similar to each other. Supplementary Fig. 2c shows the topic distributions within these newly derived subphenotypes. Finally, Supplementary Fig. 2d quantifies the overlap between the subphenotypes identified from the 44 and original 137 PASC conditions, which showed that more than 90% of the patients would remain in the same subphenotype. This demonstrated the robustness of the PASC subphenotype classification.

### Subphenotype validation on the OneFlorida+ cohort

**Extended Data Fig. 4:**
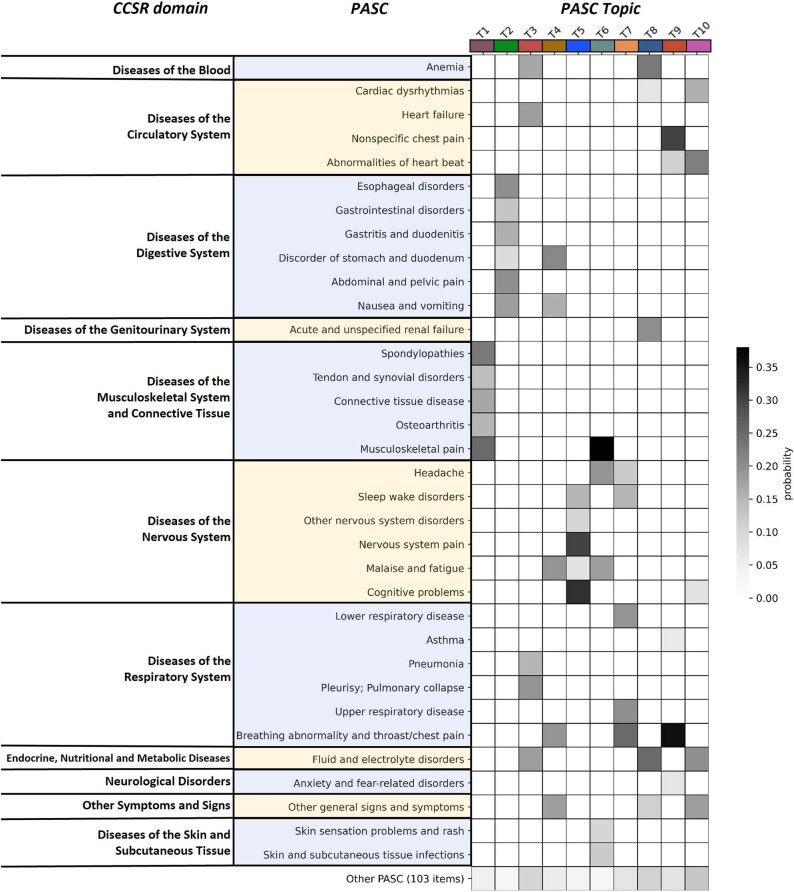
The PASC topic heatmap for the OneFlorida+ cohorts. The heatmap of PASC topics learned from the OneFlorida+ cohort. Each row denotes a potential PASC category grouped by different CCSR domains, and each column denotes a particular PASC topic. Each PASC topic is characterized by a unique post-acute incidence probability distribution over all 137 individual potential PASC categories. Source data

**Extended Data Fig. 5:**
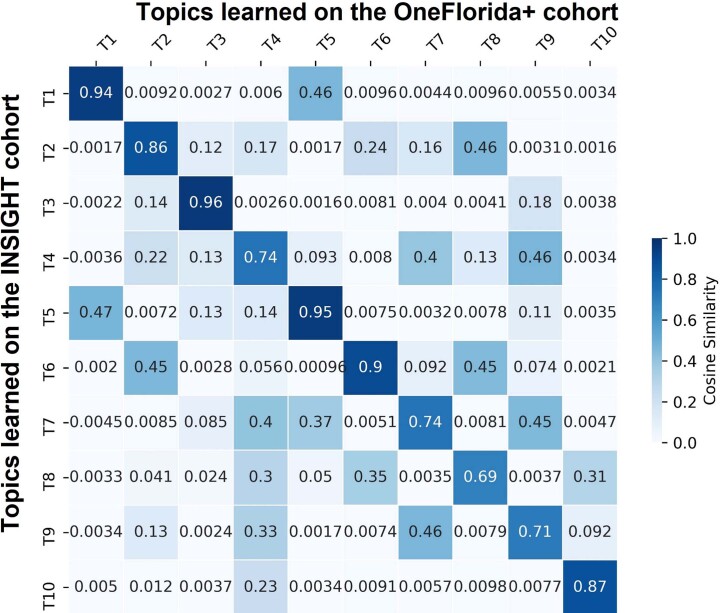
The overlap of topics between the INSIGHT and OneFlorida+ cohorts. The similarity of topics between INSIGHT and OneFlorida+ cohorts, which were used to evaluate the overlap of topics learned on two cohorts. Source data

We repeated the same subphenotyping process shown in Fig. 1 on the OneFlorida+ cohort. Extended Data Fig. 4 displays the heat map of all learned potential PASC topics, where we see topics concentrated in conditions of the musculoskeletal system (T1), digestive system (T2), nervous system (T5) and topics mixed with respiratory system conditions and blood/circulatory system conditions (T3) as well as headache and sleep-wake conditions (T7). Some topics also include a mixture of diagnoses. For example, T9 includes throat/chest pain as well as breathing/heartbeat abnormalities; T6 is a mixture of musculoskeletal pain, headache, malaise and fatigue and skin sensory conditions; T8 and T10 include electrolyte/fluid disorders and anemia/arrhythmias; and T4 includes a combination of sequelae involving digestive, nervous and respiratory systems. We quantitatively evaluated the pairwise similarities between the topics learned from the INSIGHT and OneFlorida+ cohorts (Methods) and visualized the results in Extended Data Fig. 5, which showed a one-to-one correspondence between the topics learned from the two cohorts.

**Extended Data Fig. 6:**
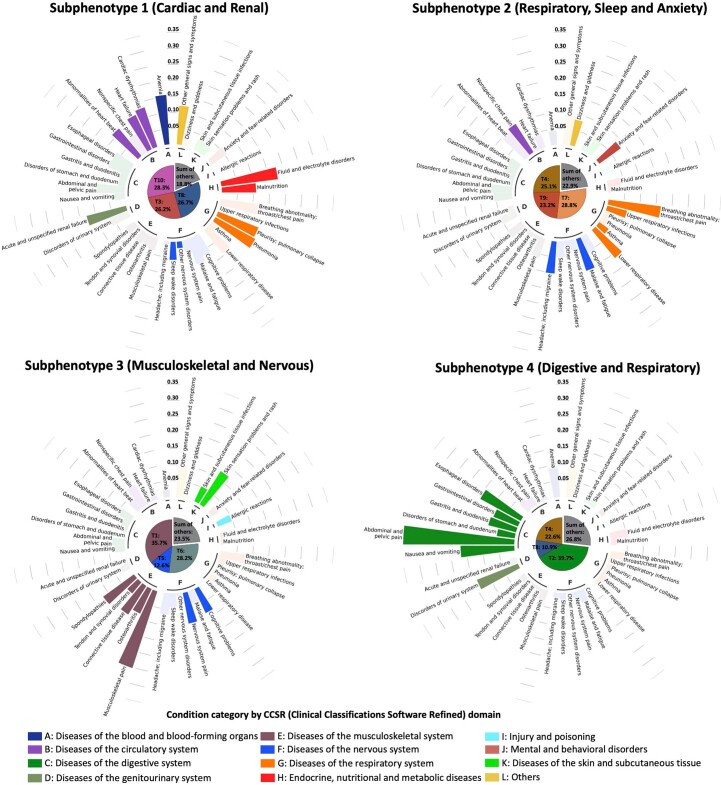
Circos plot for the OneFlorida+ subphenotypes with PASC. The incidence rates of potential PASC conditions in each subphenotype for the OneFlorida+ cohort (more numerical results are in Supplementary Table 9), where potential PASC conditions were grouped into different categories shown in different colored bars outside the center pie chart. A condition with the highest incidence rate is highlighted in the subphenotype. The center pie chart of each subphenotype shows the mean topic proportions of the patients it included, and the colors and meanings of the topic indices can be referred to Extended Data Fig. 4. Source data

**Extended Data Fig. 7:**
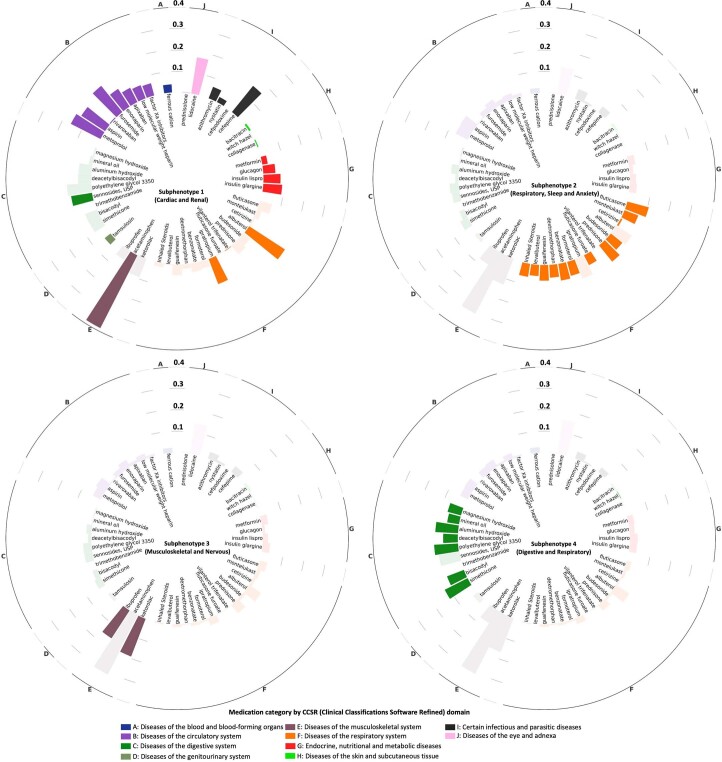
Circos plot for the OneFlorida+ subphenotypes with newly incident medications. The prevalence of incident prescriptions of medications in the post-acute infection period for each subphenotype on the OneFlorida+ cohort, where medications are grouped into different categories shown by different colors. For one of the medications, if it is most prevalent in one subphenotype, we highlighted it in this subphenotype. Source data

Extended Data Figs. 6 and 7 plot the incidence rates of PASC conditions and medication prescriptions across different subphenotypes in the post-acute infection period in the OneFlorida+ cohort, with mode details shown in Supplementary Tables 8 and 9. The results show that Subphenotype 1 was dominated by incidental cardiac and renal conditions, which included 25.43% of the patients who were older (with a median age of 62.0 years and IQR (49.0–74.0)), with highest proportion of males (46.93%, compared to 38.29% for the overall population) and highest rates of hospitalization (57.34%, compared to 36.69% overall), mechanical ventilation (8.57%, compared to 3.39% overall) and critical care admission (12.52%, compared to 6.07% overall) in the acute phase of COVID-19. This subphenotype was associated with a higher prevalence of underlying conditions and more new prescriptions for medications treating circulatory system, blood and endocrine conditions. Subphenotype 2 was dominated by incidental respiratory conditions, sleep disorders and anxiety and was the largest subphenotype, containing 5,281 (38.48%) patients with a median age of 47.0 years (IQR (33.0–61.0)). This subphenotype had a higher prevalence of respiratory conditions at baseline, including chronic obstructive pulmonary disease, pneumonia and upper respiratory tract conditions, and had higher post-acute infection incident prescriptions for respiratory medications. Subphenotype 3 was dominated by incident conditions with musculoskeletal and nervous systems. It included 3,205 (23.35%) patients with a median age of 48.0 years (IQR (33.0–61.0)) and had the lowest hospitalization rate in the acute phase (27.8%). This subphenotype had a higher prevalence of baseline musculoskeletal and connective tissue conditions and asthma and more new prescriptions for pain medications, including ketorolac and ibuprofen, in the post-acute infection phase. Subphenotype 4 was dominated by incidental digestive and respiratory conditions. It was the youngest (median age 46.0 years (IQR (32.0–60.0)) and the subphenotype with the fewest number of patients (1,748 (12.74%) patients), with the highest proportion of females (67.11%, compared to 61.70% overall) and the lowest rates of mechanical ventilation (0.97%) and critical care admission (2.8%) in the acute phase. This subphenotype was associated with a higher baseline burden of digestive system conditions and more new prescriptions for medications focused on the digestive system. These observations and characterizations were highly consistent with the subphenotypes identified from the INSIGHT cohort. In addition, we also provide the sex-disaggregated analysis results for each subphenotype with respect to patient demographics, baseline and acute infection phase medical utilizations, underlying condition prevalence and newly PASC incidence rates in Supplementary Table 10.

**Extended Data Fig. 8:**
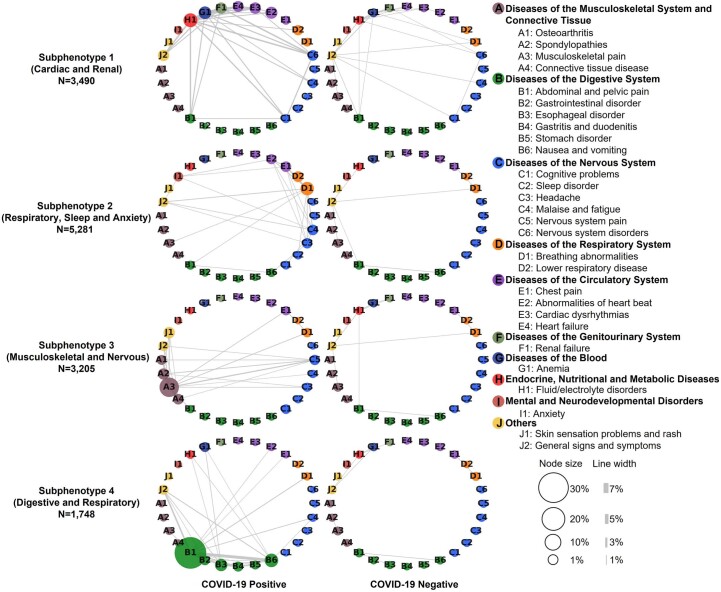
Network contrast for the OneFlorida+ cohort. Difference of the incidence patterns of selected PASC conditions (grouped by CCSR domains) in 30–180 days after COVID-19 lab test between positive and matched negative patients on the OneFlorida+ cohort. The bubbles in each network correspond to a PASC condition with their sizes proportional to the incidence rate in the particular subphenotype or matched controls. The edge linking a pair of bubbles indicates co-incidence of the corresponding investigative conditions with its thickness proportional to the co-incidence rate, where we showed the line if the rate is larger than 1%. Source data

We also replicated the contrast analysis with matched patients who tested negative for SARS-CoV-2 infection in the OneFlorida+ cohort. Similarly to the observations in the INSIGHT cohort, more topics were needed for characterizing the patients who tested negative for SARS-CoV-2 infection (Extended Data Fig. 2b). These topics were not similar to the topics learned from the patients who tested positive for SARS-CoV-2 infection (Extended Data Fig. 3d) and had less concentration patterns (Supplementary Table 6). The contrast of co-incidence patterns of the 137 PASC conditions and 28 selected conditions are shown in Supplementary Fig. 3 and Extended Data Fig. 8, which were also highly consistent with the observations from the INSIGHT cohort.

## Discussion

Several studies have found that PASC could include a diverse set of symptoms and signs involving many organ systems^10–12^. Unlike existing research that has studied these conditions independently, we developed a data-driven framework to identify subphenotypes of SARS-CoV-2-infected patients based on newly incident signs and symptoms 30–180 days after the date of confirmed infection. With the EHR from INSIGHT and OneFlorida+ CRNs, we identified four subphenotypes dominated by new conditions of the cardiac and renal systems (Subphenotype 1); respiratory system, sleep and anxiety problems (Subphenotype 2); musculoskeletal and nervous systems (Subphenotype 3); and digestive and respiratory systems (Subphenotype 4).

In both cohorts, Subphenotypes 1 and 2 are the largest two subphenotypes. Subphenotype 1 contains 33.75% and 25.43% of the patients in INSIGHT and OneFlorida+, respectively. It includes older patients with more baseline comorbidities, greater severity of acute illness in medical utilizations and higher proportion of males^23^. Many patients in this subphenotype were confirmed with SARS-CoV-2 infection during the early pandemic (March–September 2020) when NYC was the epicenter, which may explain the observation that its size is larger for INSIGHT than OneFlorida+. Early cases had greater acute phase severity as treatment protocols were still evolving, which may explain more severe incident conditions in the post-acute infection period of these patients, possibly caused by hyperinflammation^24^. Subphenotype 2 occupies 32.75% and 38.48% of the patients in INSIGHT and OneFlorida+. It includes younger patients who had SARS-CoV-2 infection confirmed mostly during July–November 2021.

Subphenotypes 3 and 4 were less prevalent. Subphenotype 3 included musculoskeletal and neurological conditions, whereas Subphenotype 4 was associated with gastrointestinal conditions. Patients in Subphenotype 3 also displayed dermatologic conditions and had the highest rates of related conditions at baseline, including autoimmune diagnoses such as rheumatoid arthritis and allergy conditions. Patients in Subphenotype 4 had the mildest acute phase severity (for example, lowest rates of mechanical ventilation and critical care admissions).

Our results suggest that the identified subphenotypes are highly consistent across the two cohorts with distinct patient populations and geographical characteristics. These four subphenotypes also covered the major PASC conditions that have been reported from existing independent studies, such as cardiovascular^3^, respiratory^25^, neurological^26^ and gastrointestinal^27^ conditions. Our study verified the co-existence of these dominate subphenotypes and can inform focused disease areas of treatment development for PASC.

There is also an existing study on identifying Long-COVID symptom clusters with information reported from 233 patients enrolled in the All-Ireland Infectious Disease cohort^13^, whereas our study is based on the diagnosis information from the EHR of large general civilian patient populations. Some of these diagnoses were with a clear diagnostic criterion, whereas others were not. For example, the conditions in Subphenotype 1, such as heart failure, pneumonia and renal failure, were mostly with objective diagnostic criteria according to underlying disease etiologies. Many conditions in Subphenotype 2 (such as breathing abnormality and non-specific chest pain), Subphenotype 3 (such as musculoskeletal and nervous system pain) and Subphenotype 4 (such as abdominal and pelvic pain, nausea and vomiting) were more subjective to diagnose. In addition, the diagnosis of certain conditions, such as esophageal and gastrointestinal disorders in Subphenotype 4, was likely to encompass functional disorders rather than clearly defined disease etiologies. This meant that our identified subphenotypes, which separated severe COVID-19 complications (Subphenotype 1) and milder PASC conditions (Subphenotypes 2, 3 and 4) that could not be explained by alternative disease etiologies and were closer to those patient-reported symptoms. These subphenotypes would help tease out the heterogeneity of these conditions and provide guidance on patient management in practice.

Our study has several strengths. First, we adopted a TM approach to derive compact patient representations based on their co-incidence patterns across different diagnoses. Unlike other dimensionality reduction techniques, such as principal component analysis (PCA)^28^, TM is designed specifically for data samples with binary or count features^29,30^ and, thus, would be appropriate for our analysis. Second, INSIGHT and OneFlorida+ include patients from distinct geographic regions in the United States with different characteristics, allowing us to validate the robustness of the derived subphenotypes. Third, our study period (March 2020 to November 2021) covers different COVID-19 waves associated with different SARS-CoV-2 virus variants.

Our study also has limitations. First, our analysis is based on longitudinal observational patient data, which cannot explain the biological mechanisms behind PASC. Second, the PASC diagnoses that we investigated were encoded as CCSR categories, which may not reflect the co-incidence patterns of fine-grained diagnosis conditions. Third, we focused on new incidences of conditions in the post-acute infection period for patients with COVID-19 and did not evaluate pre-existing conditions that may persist or worsen due to acute SARS-CoV-2 infection. Fourth, the goal of our study was to identify potential PASC subphenotypes, and we did not conduct rigorous analysis on the predictability of these subphenotypes, which was left as a future research topic. Finally, our study period did not include the COVID-19 wave dominated by the Omicron SARS-CoV-2 variant.

In conclusion, our study dissects the complexity and heterogeneity of newly incident conditions 30–180 days after SARS-CoV-2 infection confirmation into four reproducible subphenotypes based on the EHR repositories from two large CRNs using machine learning. These findings could be useful for clinicians and health systems in developing care models to meet the needs of patients with PASC.

## Methods

### EHR data repositories

Two large-scale, de-identified, real-world EHR data warehouses were used in our analyses. Our first cohort data were based on the EHR from the INSIGHT CRN^14^, which contains the longitudinal clinical information of approximately 12 million patients in the NYC area. Our second cohort data were based on the EHR from the OneFlorida+ CRN^15^, which contains the information of nearly 15 million patients mainly from Florida and selected cities in Georgia and Alabama.

This study is part of the National Institutes of Health (NIH) Researching COVID to Enhance Recovery (RECOVER) Initiative^31^, which seeks to understand, treat and prevent the post-acute sequelae of SARS-CoV-2 infection (PASC).

### Potential PASC conditions

We systematically reviewed the literature on studies related to PASC or Long-COVID and compiled a comprehensive list of potential PASC conditions in ICD-10 codes based on the one provided in Al-Aly et al.^10^.

Then, our clinician team carefully reviewed the list and removed the codes that would not plausibly be considered as PASC in the adult population (for example, HIV, tuberculosis, infection by non-COVID causes, neoplasms, injury due to external causes, etc.) and systematically added the parent codes (for example, the first three digits of ICD-10 codes). Finally, these ICD-10 codes were mapped to 137 condition categories according to CCSR version 2022.1 as Al-Aly et al.^10^ did. We added these clarifications and provide the detailed code list in Supplementary Table 2.

### Cohort construction

**Extended Data Fig. 9:**
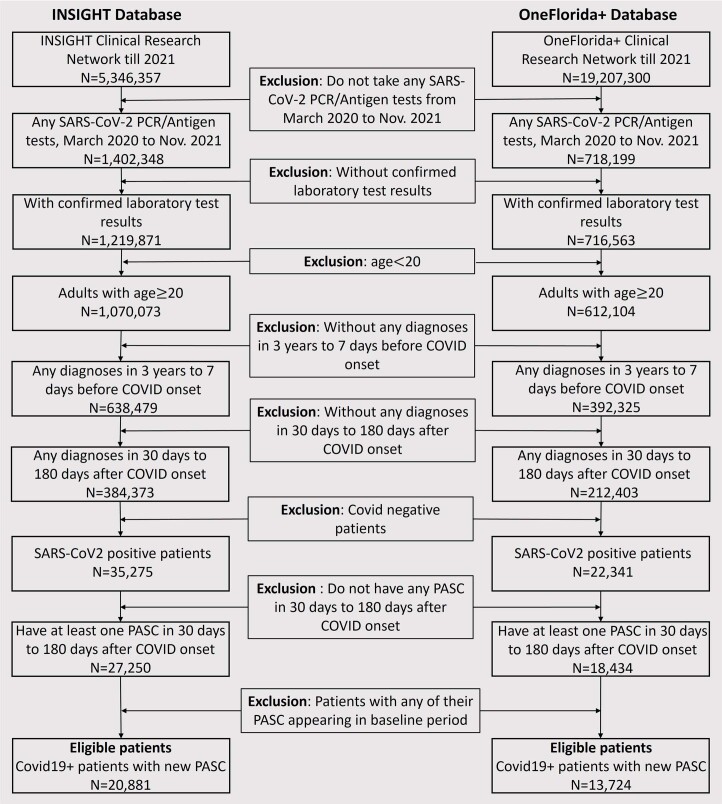
Inclusion-exclusion cascade for the INSIGHT and OneFlorida+ cohorts. Inclusion-exclusion cascades for the INSIGHT and OneFlorida+ cohorts.

For both cohorts, adult patients (age ≥20 years) with at least one SARS-CoV-2 polymerase chain reaction (PCR) or antigen laboratory test (Supplementary Table 11) between 1 March 2020 and 30 November 2021 were included. We further required these patients to have at least one diagnosis code in the baseline period and at least one diagnosis 30–180 days after index date to confirm healthcare utilization capture. Then, we chose the patients who had at least one positive test and had at least one potential PASC condition in the follow-up (or post-acute infection) period. We further made sure that those potential PASC conditions were new incidences in the follow-up period by excluding patients who had any of them in both baseline and follow-up periods. The overall inclusion–exclusion cascade is shown in Extended Data Fig. 9, and the relevant definitions are provided below.

- Index date: the date of the first COVID-19-positive test.
- Baseline period: from 3 years to 1 week before the index date.
- Follow-up (post-acute infection) period: from 31 days after the index date to the day of documented death, the last record in the database, 180 days after baseline (we followed the definition provided by the NIH and the Centers for Disease Control and Prevention (CDC)^10,22,31,32^) or the end of our observational window (30 November 2021), whichever came first.

### Statistical analysis

#### TM

We use binary vectors $\left\{ {{{{\boldsymbol{x}}}}_n} \right\}_{n = 1}^N$ to represent the patients, where *n* is the patient index the *i*-th element of ***x***_*n*_, or ***x***_*n*_(*i*) = 1 if the *i*-th potential PASC condition appears in the post-acute infection period of the *n*-th patient’s EHR, otherwise ***x***_*n*_(*i*) = 0. Therefore, each ***x***_*n*_ is a 137-dimensional binary vector (step 1 in Fig. 1). TM^19^ was then applied on these vectors to learn a set of potential PASC topics. PASC topics in this context refers to a group of conditions that present together based on their incident probabilities. Specifically, assume that each patient can be represented as a mixture of *K* latent PASC topics **Φ**∈*R*^*D*×*K*^, where *D* = 137 is the number of unique PASC conditions and *K* is the total number of topics. Each topic ***φ***_k_ ($k:1 \le k \le K$ is the index of the topic) can be viewed as a set of PASC that are more likely to be co-incident in the post-acute infection period of a particular patient (step 2 in Fig. 1). Then, for each patient, TM infers the mixture memberships ***θ***_*n*_∈*R*^*K*^, also called topic proportions or topic loadings, as the new representation for each patient (step 3 in Fig. 1). A patient with higher loading value on a particular topic indicates that he/she suffers from more co-incident condition patterns from this topic. In other words, TM transforms the representations of each patient from the original 137-dimensional binary space ***x***_*n*_ to the low-dimensional continuous PASC topic space ***θ***_*n*_, which will be leveraged further for subphenotype identification through clustering later (step 4 in Fig. 1). Specifically, we used Poisson factor analysis (PFA)^17^ as the concrete TM method, which generates ***x***_*n*_ as follows.

- Draw a topic proportion ***θ***_*n*_ for the *n*-th patient from a Gamma distribution ***θ***_*n*_ ~ *Gamma*(1,1);
- Draw the *k*-th PASC topic from a Dirichlet distribution ***φ***_*k*_ ~ *Dirichlet*(0.01), $k = 1, \cdots ,K$;
- Draw a binary vector *x*_*n*_ from the Bernoulli distribution by Bernoulli–Poisson link^33–36^:

$${{{\boldsymbol{x}}}}_n = 1\left( {{{{\boldsymbol{u}}}}_n \ge 1} \right),{{{\boldsymbol{u}}}}_{{{\boldsymbol{n}}}} \sim Poisson\left( {{{{\bf{\Phi }}}}{{{\mathbf{\theta }}}}_n} \right);$$

where $1\left( \cdot \right)$ is an indicator function representing ***x***_*n*_ = 1 if ***u***_*n*_ ≥ 1 and ***x***_*n*_ = 0 if ***u***_*n*_ = 0. The input of the PFA model is the observed patient binary vector $\left\{ {{{{\boldsymbol{x}}}}_n} \right\}_{n = 1}^N$. The learnable parameters of the PFA model are the topic proportion vectors $\left\{ {{{{\mathbf{\theta }}}}_n} \right\}_{n = 1}^N$ and the topic matrix **Φ**. Because the PFA is a fully conjugated probabilistic model, we trained it by Gibbs sampling with the conditional posterior of the learnable parameters, whose specific equations can be found in Zhou et al.^17^. We used the Python package Pydpm version 3.0.1 (https://pypi.org/project/pydpm/) to train the model and released the code.

For PFA, we imposed Dirichlet as the prior distribution for *k*-th PASC topic so that it lies in a probability simplex space (elements in ***φ***_*k*_ represent the probability of each PASC appearing in *k*-th topic). Compared to another popular TM method—latent Dirichlet allocation (LDA)^29^ that imposes Dirichlet distribution on the topic proportions ***θ***_*n*_—PFA uses Gamma distribution that can obtain more separable compressed representations^17,35^, which is useful for identifying more meaningful subphenotypes.

The number of topics, *K*, is an important parameter in TM. To determine an optimal *K* based on the data, we used two metrics: data likelihood and topic coherence^37^. Data likelihood is used to evaluate the fitness of the model on the current dataset, where the larger value indicates better fitness. Topic coherence is used to evaluate the relevance of our learned PASC topics to the investigative condition list, where the value is from 0 to 1, and higher value indicates better coherence. Detailed calculations of these two metrics are provided below.

##### Data likelihood

In PFA, to model the binary PASC vector, we used the Bernoulli–Poisson link as:

$${{{\boldsymbol{x}}}}_n = 1\left( {{{{\boldsymbol{u}}}}_n \ge 1} \right),{{{\boldsymbol{u}}}}_{{{\boldsymbol{n}}}} \sim Poisson\left( {{{{\bf{\Phi }}}}{{{\mathbf{\theta }}}}_n} \right);$$

where ***x***_*n*_ is the binary PASC vector, **Φ** is the topic matrix, ***θ***_*n*_ is the topic proportion vector and ***u***_***n***_ is the latent variable that links the binary observation and topic representation. According to the property of Bernoulli–Poisson link, ***u***_***n***_ can be marginalized out, and then one can obtain a Bernoulli likelihood as

$${{{\boldsymbol{x}}}}_n \sim Ber\left( {1 - {{{\mathrm{exp}}}}^{ - {{{\bf{\Phi }}}}{{{\mathbf{\theta }}}}_n}} \right).$$

Given $\{ {{{\boldsymbol{x}}}}_n\} _{n = 1}^N$, after learning **Φ** and $\{ {{{\mathbf{\theta }}}}_n\} _{n = 1}^N$, we can calculate the Bernoulli data likelihood. In Extended Data Fig. 2, we show the mean (average of the number of patients) data log-likelihood:

$$\frac{1}{N}\mathop {\sum}\limits_{n = 1}^N {\mathop {\sum}\limits_{v = 1}^V {\hat x_{n,v}log\left( {x_{n,v}} \right) + \left( {1 - \hat x_{n,v}} \right)log\left( {1 - x_{n,v}} \right)} } ,$$

where *N* is the number of patients and *V* is the total number of PASC, and $\hat x_{n,v}$ is the averaged reconstruction item. Because each iteration of Gibbs sampling provides only a point estimation, to obtain the likelihood of the distribution estimation in practice we collect the parameters of every iteration after burn-in step (500 burn-in steps in our experiment) and then take the average of them (Zhou et al.^17^), defined as ${{{\hat{\boldsymbol x}}}}_n = \frac{1}{Q}\mathop {\sum}\nolimits_{q = 1}^Q {1 - {{{\mathrm{exp}}}}^{ - {{{\mathbf{\Phi }}}}^{\left( {{{\boldsymbol{q}}}} \right)}{{{\mathbf{\theta }}}}_n^{\left( q \right)}}}$, where *Q* is the total number of collected parameters (*Q* = 500 in our experiment).

##### Topic coherence

Topic coherence is an important metric to evaluate the quality of topics based on the input data. It is measured based on a sliding window (in our case, the length of the window is the total number of PASCs), and then one can calculate the normalized pointwise mutual information (NPMI) between input data and the learned topics. We used the Python package GENSIM (https://radimrehurek.com/gensim/) to calculate the topic coherence.

The results are shown in Extended Data Fig. 2. From this figure, we can see that more topics can provide higher data likelihood because we have more topics to represent the original PASCs. However, more topics may bring down topic coherence, which suggests the redundancy between the newly added ones and the old ones. With these considerations, we set the final number of topics as 10 for both INSIGHT and OneFlorida+ cohorts, as it achieved the best topic coherence and reasonable data likelihood (we do not want the data likelihood to be too perfect, as that may suggest overfitting).

As TM is a probabilistic process, we want to guarantee the robustness of the identified topics. To achieve this goal, we first did 1,000 bootstrapping (randomly sample the same number of patients with replacement) to learn topics. Then, according to the importance of each topic (mean topic loadings over all patients), we reordered the topics and calculated the cosine similarity among all topics from each pair of bootstrapping:

$$S_{pq} = \frac{1}{{1000}}\mathop {\sum}\limits_{i = 1}^{1000} {\frac{1}{{999}}} \mathop {\sum}\limits_{j = 1,j \ne i}^{1000} {cos} \left( {{{{\mathbf{\varphi }}}}_p^{\left( i \right)},{{{\mathbf{\varphi }}}}_q^{\left( j \right)}} \right),$$

where ${{{\mathbf{\varphi }}}}_p^{\left( i \right)}$ is the *p*-th topic vector learned from the *i*-th bootstrapped samples, and *S*_*pq*_ is the similarity between the *p*-th topic and the *q*-th topic. Extended Data Fig. 3a,b demonstrates the heat map of the similarity matrix with *S*_*pq*_ as its (*p*,*q*)-th entry, from which we can clearly observe a darker diagonal line, which indicates a high similarity between the topics learned from different bootstrapped samples and, thus, implies that the learned topics are robust.

We also quantitatively evaluated the consistency between the two set of topics learned from different cohorts. Specifically, denoting the topic matrices learned from the two cohorts as **Φ**^(1)^ and **Φ**^(2)^, then we can evaluate the consistency between the *i*-th topic in cohort 1 and the *j*-th topic in cohort 2 as the cosine similarity between their corresponding topic vector ${{{\mathbf{\varphi }}}}_i^{\left( 1 \right)}$ and ${{{\mathbf{\varphi }}}}_j^{\left( 2 \right)}$ as:

$$S_{ij} = cos\left( {{{{\mathbf{\varphi }}}}_i^{\left( 1 \right)},{{{\mathbf{\varphi }}}}_j^{\left( 2 \right)}} \right),i,j = 1, \cdots ,K,$$

Finally, the heat map of the topic consistency matrix with *S*_*ij*_ as its (*i*,*j*)-th entry was shown in Extended Data Fig. 5, from which we can clearly observe darker diagonal values, which suggests high consistency between the two sets of topics. In addition, the topic consistency learned from the positive and negative cohorts is provided as results in Extended Data Fig. 3c,d, which demonstrates low consistency between two sets of topics from the positive and negative cohorts.

We calculated the entropy of each topic to quantitatively evaluate the concentration patterns of each topic. Specifically, because each topic is a probabilistic distribution over the PASC conditions, we can use the following definition of entropy to evaluate whether each topic is closer to a uniform distribution or concentrates on some related PASC.

$$H\left[ {{{{\mathbf{\varphi }}}}_k} \right] = - \mathop {\sum}\limits_{i = 1}^{137} {\varphi _{ki} \times log_2\varphi _{ki}} ,$$

where *φ*_*ki*_ is the *i*-th element in ***φ***_*k*_. In our case, *H*[***φ***_*k*_] should be in a range of (0, 7.0980). Smaller *H*[***φ***_*k*_] denotes that the *k*-th topic more likely concentrates on particular PASC conditions (capturing stronger co-occurrence pattern of PASC), whereas larger *H*[***φ***_*k*_] denotes that the *k*-th topic is closer to a uniform distribution. From Supplementary Table 6, we observed that those topics learned from the positive cohort had lower entropy values than those learned from the negative cohort.

#### Subphenotyping through clustering

**Extended Data Fig. 10:**
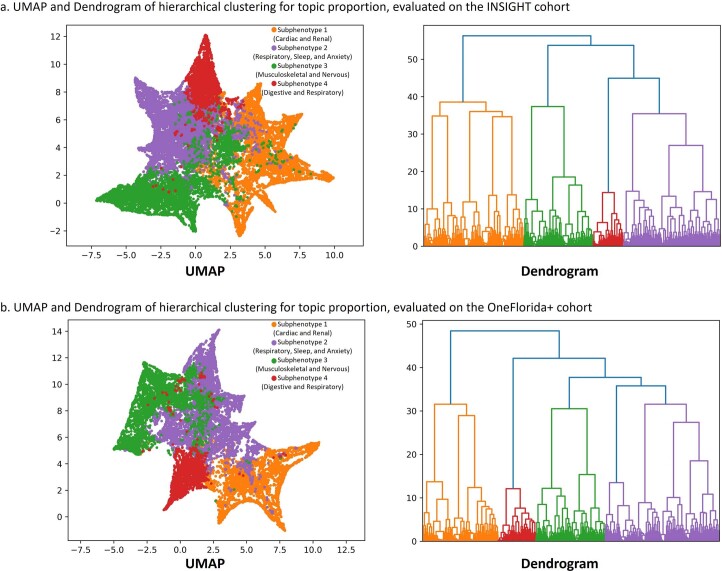
UMAP and dendrogram for the INSIGHT and OneFlorida+ cohorts. Uniform Manifold Approximation and Projection (UMAP) embedding results and dendrograms for the INSIGHT and OneFlorida+ cohorts. Source data

With the learned *K*-dimensional topic loading vector for *n*-th patient ***θ***_*n*_, we applied the hierarchical agglomerative clustering method with Euclidean distance calculation and Ward linkage criterion^38^ to derive subphenotypes as patient clusters. For determining the optimal number of clusters (subphenotypes), according to Su et al.^39^ and Xu et al.^40^, we applied the NbClust R package^41^, which includes 21 cluster indices to evaluate the quality of clusters. With the patients from the INSIGHT and OneFlorida+ CRN, 13 and 12 out of the 21 indices agreed that four is the optimal number of clusters (Supplementary Table 12). Through majority voting, we set the number of clusters as four in both two cohorts. Extended Data Fig. 10 demonstrates the uniform manifold approximation and projection (UMAP) embeddings^42^ and dendrogram of these clusters for both cohorts.

We also examined the robustness of the identified clusters on both cohorts. Specifically, for each cohort, we used the subphenotypes derived from all patients as references, so that the subphenotype index for the *n*-th patient is denoted as *y*_*n*_. Then, we ran 1,000 bootstrapping (randomly choose 80% patients from the cohort denoted as set ${{\Omega }}_{{{\mathrm{i}}}},i = 1, \cdots ,1000$) to learn the topic model and then derive subphenotypes with the same procedure as described above. For each bootstrapped sample set *i*, we can obtain another subphenotype index for *n*-th patient from Ω_i_ as $\hat y_{n,i}$. We calculated the mean and 95% confidence interval (CI) of Adjusted Rand Index (ARI) score and normalized mutual information (NMI) between the clustering results on bootstrapped sample sets and reference as 0.902 (95% CI: 0.863–0.927) and 0.937 (95% CI: 0.908–0.952) for the INSIGHT cohort and 0.914 (95% CI: 0.907–0.929) and 0.950 (95% CI: 0.936–0.968) for the OneFlorida+ cohort, which suggests that the identified clusters are highly robust.

#### Comparison with patients without SARS-CoV-2 infection

We compared the co-incidence patterns of the investigative conditions in the follow-up periods for patients with SARS-CoV-2 infection testing positive and negative. The patients who tested negative for SARS-CoV-2 all had negative results from lab tests between March 2020 and November 2021, and there were no documented COVID-19-related diagnoses during this time period. The index date for each individual patient in the non-infected group is defined as the date of the first (negative) lab test.

To make fair comparisons, we performed similarity matching to identify appropriate negative patients for each positive patient based on the following hypothetical confounding variables.

- Demographics: age, gender, race and ethnicity, where age was binned into different groups (20–<40 years, 40–<55 years, 55–<65 years, 65–<75 years, 75–<85 years and 85+ years).
- The ADI (ten rank bins of national ADI) for capturing the socioeconomic disadvantage of patients’ neighborhood^18^.
- Index date for considering the effect of different stages of the pandemic, which was binned into different time intervals (March–June 2020, July–October 2020, November 2020 to February 2021, March–June 2021 and July–November 2021).
- Medical utilizations measured by numbers of inpatient, outpatient and emergency encounters in the baseline period (binned into 0 visit, 1 or 2 visits, 3 or 4 visits and 5+ visits for each encounter type).
- Coexisting conditions, including comorbidities and medications based on a tailored list of the Elixhauser comorbidities^43^. We defined the patient having a particular condition if he/she had at least two related records during the baseline period.

For identifying the negative controls for each patient in a particular subphenotype, we first required exact match for confounders of demographics, ADI and index date to obtain an initial set, and then we performed robust PS matching on other hypothetical confounders to rank the patients in the initial set, and we finally picked the top two. We used standardized mean difference (SMD) to quantify the goodness-of-balance of confounders between two groups $SMD\left( {X_1,X_0} \right) = \frac{{\left| {E\left[ {X_1} \right] - E\left[ {X_0} \right]} \right|}}{{\root {2} \of {{\left( {Var\left( {X_1} \right) + Var\left( {X_0} \right)} \right)/2}}}}$, where SMD < 0.2 is the threshold to examine whether this confounder is balanced^44^. On both INSIGHT and OneFlorida+ cohorts, we found that all confounders on all subphenotypes were balanced.

#### Shortening the PASC condition list with high-dimensional PS adjustment

We leveraged our high-dimensional PS adjustment pipeline^20,21^ to identify the potential PASC conditions that showed significantly higher risk during 30–180 days after lab-confirmed SARS-CoV-2 infection compared to patients who tested negative for SARS-CoV-2, after adjusting for a comprehensive list of hypothetical confounders, including demographics, baseline medical utilizations and comorbidities, and severity during the acute infection phase. More details can be foud in Zang et al.^21^, and the results on different PASC conditions are provided in Supplementary Table 7. We selected a particular PASC condition if either of the following two conditions was satisfied.

- Hazard ratio (positive over negative) was larger than 1, and *P* value was smaller than 0.05/137.
- Hazard ratio is larger than 1, and *P* value is smaller than 0.05, and it is reported or suspected in the PASC-related works^10,22^.

With these two rules, we were able to identify 44 potential PASC conditions, which are provided in Supplementary Table 2.

All statistical analysis was performed with Python 3.7—Python package scikit-learn-0.23.2, numpy-1.16.5, umap-learn-0.5.1, Pydpm-3.0 and scipy-1.7.3 for machine learning models.

### Ethics approval

Use of the INSIGHT data was approved by the institutional review board (IRB) of Weill Cornell Medicine following protocol 21–10–95–380 with the title ‘Adult PCORnet-PASC Response to the Proposed Revised Milestones for the PASC EHR/ORWD Teams (RECOVER)’. Use of the OneFlorida+ data for this study was approved under University of Florida IRB number IRB202001831. All EHRs used in this study were appropriately de-identified, and, thus, no informed consents from patients were obtained.

### Reporting summary

Further information on research design is available in the Nature Portfolio Reporting Summary linked to this article.

## Online content

Any methods, additional references, Nature Portfolio reporting summaries, source data, extended data, supplementary information, acknowledgements, peer review information; details of author contributions and competing interests; and statements of data and code availability are available at 10.1038/s41591-022-02116-3.

## Supplementary information

Supplementary Information Supplementary Figs. 1–3

Reporting Summary

Supplementary Tables 1–12 All Supplementary Tables

## Data Availability

Information of the INSIGHT CRN is provided at https://insightcrn.org/, and INSIGHT data (https://onefloridaconsortium.org/) are made available to researchers with an approved study protocol at https://nyc-cdrn.atlassian.net/servicedesk/customer/portal/2/group/6/create/16. Information of the OneFlorida+ CRN is provided at https://onefloridaconsortium.org/, and OneFlorida+ data are made available to researchers with an approved study protocol at https://onefloridaconsortium.org/front-door/prep-to-research-data-query/. For questions regarding INSIGHT, email: insightcrn@med.cornell.edu. For questions regarding OneFlorida+, email: OneFloridaOperations@health.ufl.edu. Source data are provided with this paper.

## Source data

Source Data Fig. 2 Statistical Source Data
Source Data Fig. 3 Statistical Source Data
Source Data Fig. 4 Statistical Source Data
Source Data Extended Data Fig. 1 Statistical Source Data
Source Data Extended Data Fig. 2 Statistical Source Data
Source Data Extended Data Fig. 3 Statistical Source Data
Source Data Extended Data Fig. 4 Statistical Source Data
Source Data Extended Data Fig. 5 Statistical Source Data
Source Data Extended Data Fig. 6 Statistical Source Data
Source Data Extended Data Fig. 7 Statistical Source Data
Source Data Extended Data Fig. 8 Statistical Source Data
Source Data Extended Data Fig. 10 Statistical Source Data

## Code availability

Our codes are available at https://github.com/haozhangWCM/Subphenotyping-for-PASC.
