# Supplementary material for: Data-driven identification of post-acute SARS-CoV-2 infection subphenotypes
Source: Nat Med. 2022 Dec 1;29(1):226–35. doi: 10.1038/s41591-022-02116-3 (PMC9873564; doi:10.1038/s41591-022-02116-3)
Supplement: Supplementary file 1 — Supplementary Figs. 1–3 [file 41591_2022_2116_MOESM1_ESM.pdf]

nature medicine

Article

https://doi.org/10.1038/s41591-022-02116-3

Data-driven identification of post-acute SARS-CoV-2 infection subphenotypes

In the format provided by the authors and unedited

Table-of-contents

Supplementary Figure 1……1

Supplementary Figure 2……2

Supplementary Figure 3……3

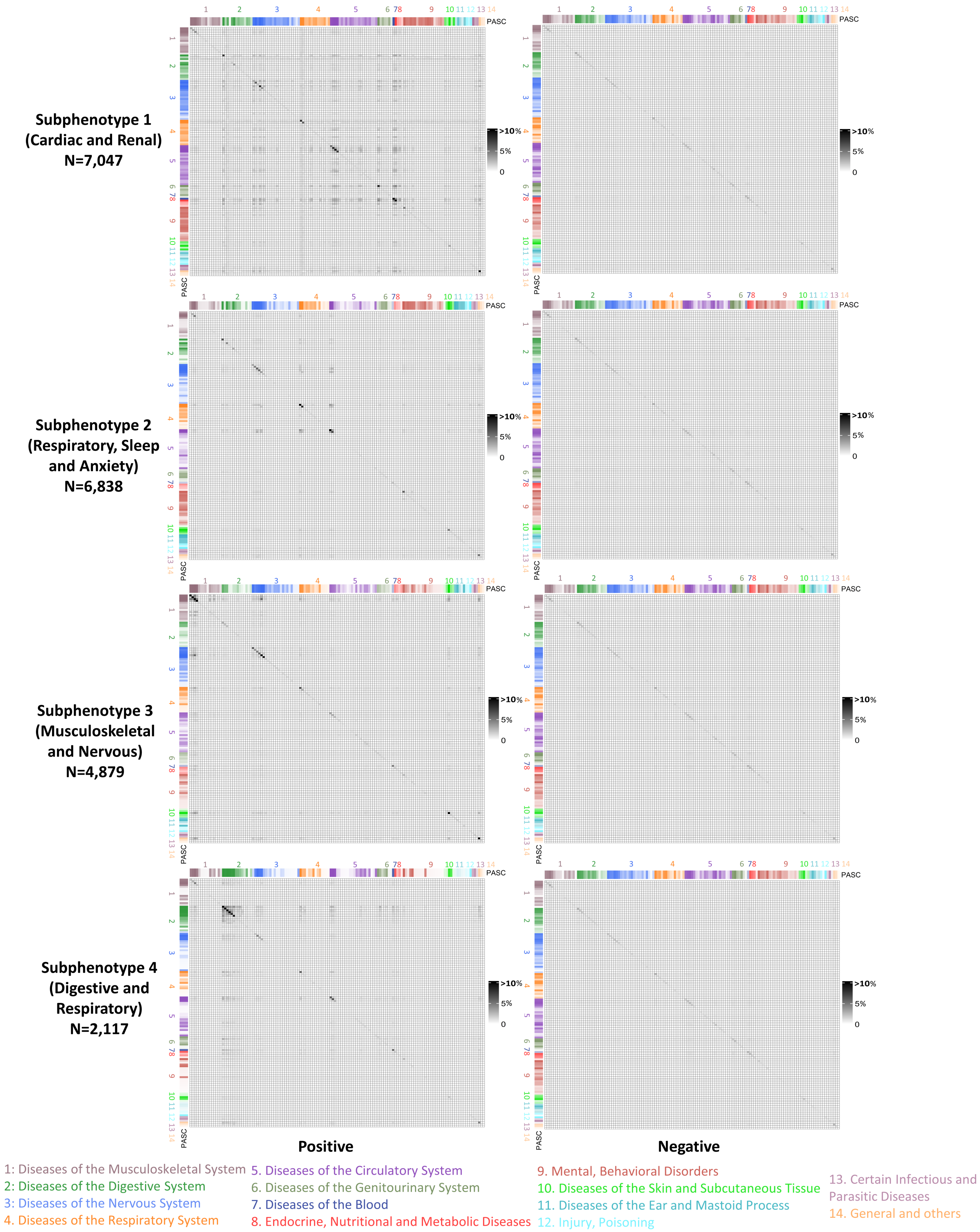

Supplementary Figure 1. Difference of the incidence patterns of all PASC conditions on the INSIGHT cohort (extension of Figure 4). The order of PASC follows the one provided in the page "137 PASC list" in the Supplementary Table 2.

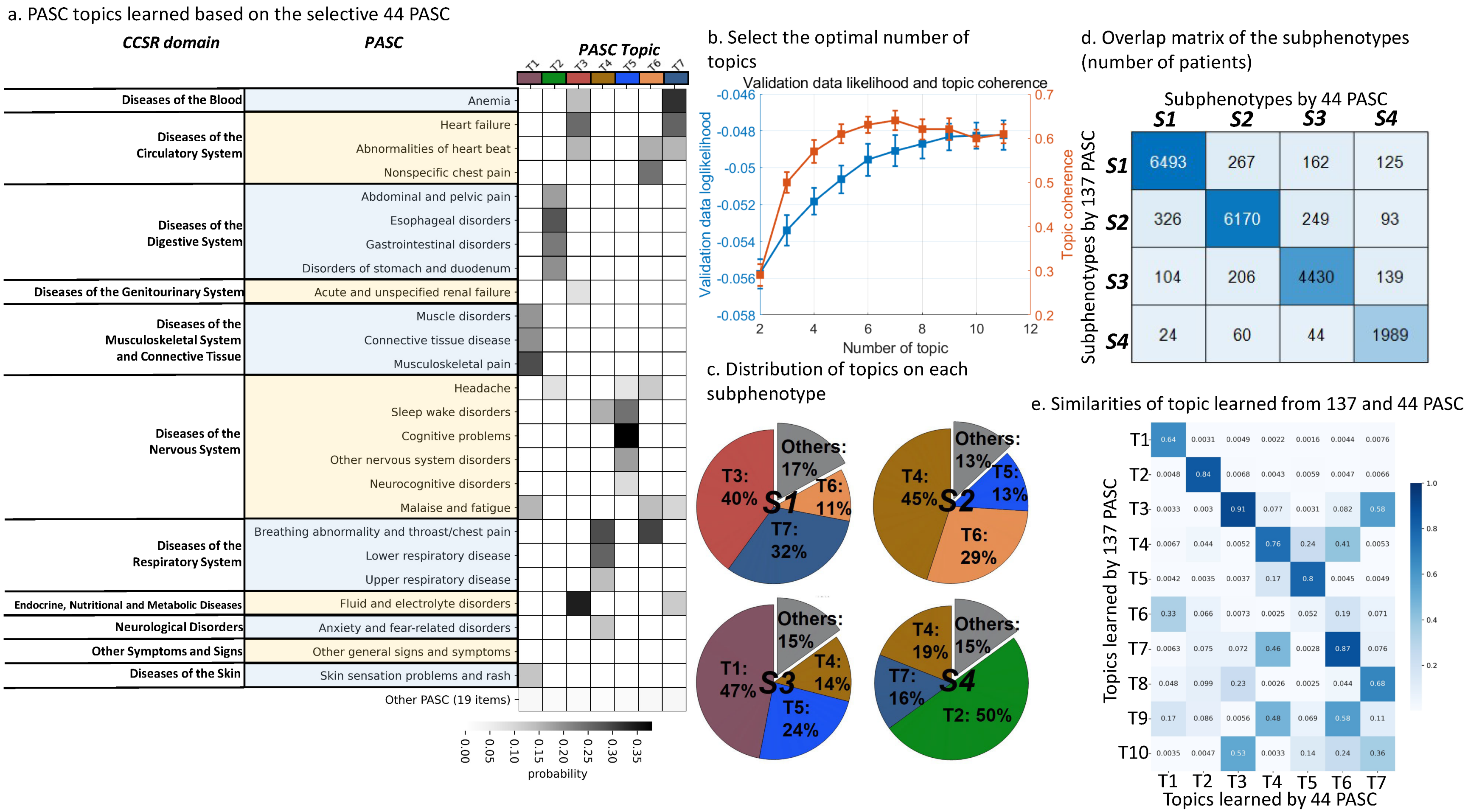

Supplementary Figure 2. We learned topics and derived subphenotypes based on the 44 selective PASC in the INSIGHT cohort. (a) shows seven topics learned based on the 44 selective PASC. (b) shows the procedure of selection optimal number of topics, where the error bar is obtained based on 5-fold cross-validation (n_training=16705, n_validation=4176), and data are presented as mean values +/- SD. (c) shows the distributions of mean topic proportions on each subphenotype. (c) shows the mean topic proportions on each subphenotype; (d) shows the overlap matrix of subphenotypes, where the number in i-th row j-th column denotes the number of patients who belongs to the i-th subphenotype derived by 137 PASC but belongs to the j-th subphenotype derived by 44 PASC. (d) shows the similarities of topics learned from 137 PASC and 44 PASC.

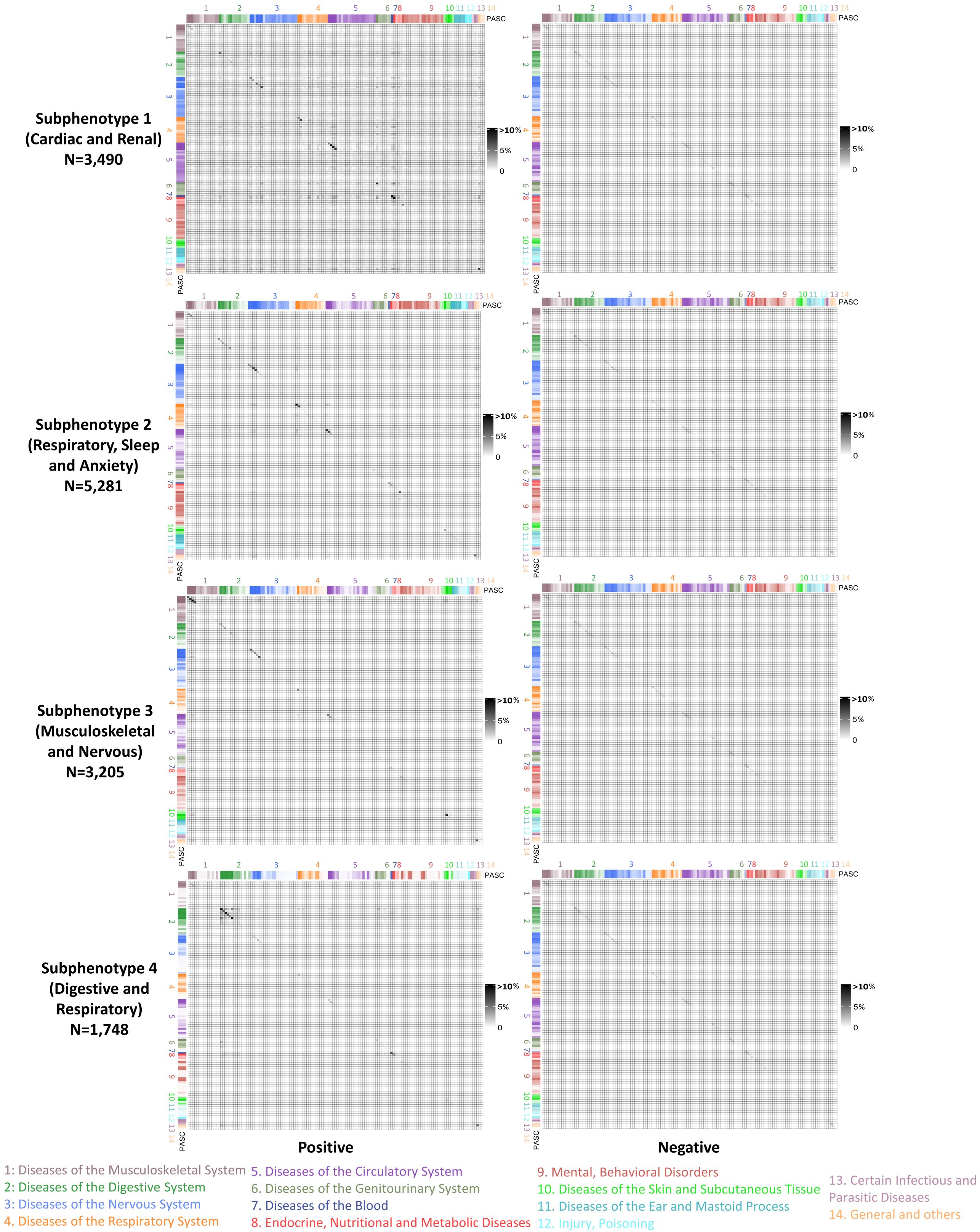

Supplementary Figure 3. Difference of the incidence patterns of all PASC conditions on the OneFlorida+ cohort (extension of Extended Data Figure 8). The order of PASC follows the one provided in the page "137 PASC list" in the Supplementary Table 2.
