# Supplementary material for: Data-driven identification of post-acute SARS-CoV-2 infection subphenotypes
Source: Nat Med. 2022 Dec 1;29(1):226–35. doi: 10.1038/s41591-022-02116-3 (PMC9873564; doi:10.1038/s41591-022-02116-3)
Supplement: Supplementary file 2 — Reporting Summary [file 41591_2022_2116_MOESM2_ESM.pdf]

Corresponding author(s): Fei Wang

Last updated by author(s): Oct 20, 2022

Reporting Summary

Nature Portfolio wishes to improve the reproducibility of the work that we publish. This form provides structure for consistency and transparency in reporting. For further information on Nature Portfolio policies, see our Editorial Policies and the Editorial Policy Checklist.

Statistics

For all statistical analyses, confirm that the following items are present in the figure legend, table legend, main text, or Methods section.

n/a	Confirmed	
☐	☒	The exact sample size (*n*) for each experimental group/condition, given as a discrete number and unit of measurement
☐	☒	A statement on whether measurements were taken from distinct samples or whether the same sample was measured repeatedly
☐	☒	The statistical test(s) used AND whether they are one- or two-sided<br>*Only common tests should be described solely by name; describe more complex techniques in the Methods section.*
☐	☒	A description of all covariates tested
☐	☒	A description of any assumptions or corrections, such as tests of normality and adjustment for multiple comparisons
☐	☒	A full description of the statistical parameters including central tendency (e.g. means) or other basic estimates (e.g. regression coefficient) AND variation (e.g. standard deviation) or associated estimates of uncertainty (e.g. confidence intervals)
☐	☒	For null hypothesis testing, the test statistic (e.g. *F*, *t*, *r*) with confidence intervals, effect sizes, degrees of freedom and *P* value noted<br>*Give P values as exact values whenever suitable.*
☒	☐	For Bayesian analysis, information on the choice of priors and Markov chain Monte Carlo settings
☒	☐	For hierarchical and complex designs, identification of the appropriate level for tests and full reporting of outcomes
☒	☐	Estimates of effect sizes (e.g. Cohen's *d*, Pearson's *r*), indicating how they were calculated

Our web collection on statistics for biologists contains articles on many of the points above.

Software and code

Policy information about availability of computer code

Data collection	SAS Enterprise Guide Version 7.1 was used to collect the data for this study. ICD-10 diagnosis codes were classified into 137 diagnostic categories based on the Clinical Classification Software Refined (CCSR) version 2021.1 (Healthcare Cost and Utilization Project, Agency for Healthcare Research and Quality).
Data analysis	For reproducibility, our codes are available at https://github.com/haozhangWCM/Subphenotyping-for-PASC. We used Python 3.7, python package scikit-learn-0.23.2, numpy-1.16.5, umap-learn-0.5.1, and scipy-1.7.3 for machine learning models.

For manuscripts utilizing custom algorithms or software that are central to the research but not yet described in published literature, software must be made available to editors and reviewers. We strongly encourage code deposition in a community repository (e.g. GitHub). See the Nature Portfolio guidelines for submitting code & software for further information.

Data

Policy information about availability of data

All manuscripts must include a data availability statement. This statement should provide the following information, where applicable:

- Accession codes, unique identifiers, or web links for publicly available datasets
- A description of any restrictions on data availability
- For clinical datasets or third party data, please ensure that the statement adheres to our policy

The information of the INSIGHT CRN is provided at https://insightcrn.org/ and INSIGHT data https://onefloridaconsortium.org/ are made available to researchers

with approved study protocol at https://nyc-cdrn.atlassian.net/servicedesk/customer/portal/2/group/6/create/16. The information of the OneFlorida+ CRN is provided at https://onefloridaconsortium.org/ and OneFlorida+ data are made available to researchers with approved study protocol at https://onefloridaconsortium.org/front-door/prep-to-research-data-query/. Questions regarding INSIGHT can email insightcrn@med.cornell.edu. Questions regarding OneFlorida+ can email OneFloridaOperations@health.ufl.edu.

Human research participants

Policy information about studies involving human research participants and Sex and Gender in Research.

Reporting on sex and gender

This is a retrospective secondary analysis of two electronic health record (EHR) cohorts. Summary statistics on sex distributions within different cohorts were reported in Table 1. Summary statistics on sex distributions within different subphenotypes were reported in Table 2.

Population characteristics

Our study included 20,881 patients from the INSIGHT clinical research network (CRN) and 13,724 patients from the OneFlorida+ CRN who tested positive for SARS-CoV-2 on viral tests (see Methods for detailed inclusion-exclusion criteria). The patients within the INSIGHT cohort had a median age of 58.0 (interquartile range [IQR] [42.0-70.0]) and a median Area Deprivation Index (ADI) of 15.0 (IQR [6.0-25.0]), consisting of 12,188 (58.37%) females, 7013 (33.59%) White patients, and 4771 (22.85%) Black patients. The OneFlorida+ cohort contained patients who were younger (median age of 51.0 (IQR [35.0-65.0])), with more disadvantaged social conditions (median ADI 59.0; IQR [42.0-76.0])), and more white patients (7175; 52.28%). 33.04% of the INSIGHT CRN patients had a confirmed SARS-CoV-2 infection from March to June 2020 (compared to 8.83% of the patients from OneFlorida+). This coincided with the first wave of COVID-19 in the US when NYC was the epicenter. Patients from OneFlorida+ were more likely to test positive from July to October 2020 (26% vs. 6% of patients from INSIGHT). Table 1 in the manuscript summarized the summary statistics of the patients from the two cohorts.

Recruitment

This is a retrospective secondary analysis of EHR data and no patient recruitment activities are involved.

Ethics oversight

The use of the INSIGHT data was approved by the Institutional Review Board (IRB) of Weill Cornell Medicine following protocol 21-10-95-380 with title “Adult PCORnet-PASC Response to the Proposed Revised Milestones for the PASC EHR/ ORWD Teams (RECOVER)”. The use of the OneFlorida+ data for this study was approved under the University of Florida IRB number IRB202001831.

Note that full information on the approval of the study protocol must also be provided in the manuscript.

Field-specific reporting

Please select the one below that is the best fit for your research. If you are not sure, read the appropriate sections before making your selection.

☒ Life sciences ☐ Behavioural & social sciences ☐ Ecological, evolutionary & environmental sciences

For a reference copy of the document with all sections, see nature.com/documents/nr-reporting-summary-flat.pdf

Life sciences study design

All studies must disclose on these points even when the disclosure is negative.

Sample size

This is a retrospective secondary EHR cohort analysis leveraging two large regional clinical research networks (CRN) with in US. To achieve better precision of the study results, we have enrolled all eligible patients from the two cohorts, which included 20,881 patients from the CRN and 13,724 patients from the OneFlorida+ CRN who tested positive for SARS-CoV-2 on viral tests and had at least one new incidental diagnosis in the period of 30-180 days after SARS-CoV-2 infection. To our knowledge, this is the largest and most comprehensive study focusing on general civilian adult population.

Data exclusions

For the both cohorts, adult patients (age ≥ 20) with at least one SARS-CoV-2 polymerase-chain-reaction (PCR) or antigen laboratory test (Supplemental Table 2) between March 01, 2020 and November 30, 2021 were selected. Then we chose the patients who had at least one positive test and had at least one potential PASC conditions in the follow-up (or post-acute infection) period defined as below. We further made sure those potential PASC conditions were new incidences in the follow-up period by excluding patients who had any of them in both baseline and follow-up periods. The overall inclusion-exclusion cascade was shown in Extended Data Figure 2, and the relevant definitions are provided below.

Index date: the date of the first COVID-19 positive test.
Baseline period: from 3 years to one week prior to the index date.
Follow-up (post-acute infection) period: from 31 days after the index date to the day of documented death, last record in the database, 180 days after baseline, or the end of our observational window (Nov. 30, 2021), whichever came first.

Replication

To provide results with better accuracy and precision, we have included all eligible patients in both INSIGHT and OneFlorida+ cohorts. The main analysis was conducted on the INSIGHT cohort and a replication analysis was done on the OneFlorida+ cohort once.

Randomization

This is a retrospective analysis based on clustering and no randomization procedure was involved as no treatment effect was assessed. For the contrast analysis with COVID-19 patients, a similarity based matching procedure was performed for identifying appropriate COVID-19 patients to make fair comparisons. The matching covariates include the following

• Demographics: age, gender, race, and ethnicity, where age was binned into different groups (20-<40 years, 40-<55 years, 55-<65 years, 65-<75 years, 75-<85 years, 85+ years).
• The area deprivation index (10-rank bins of national ADI) for capturing socioeconomic disadvantage of patients' neighborhood13.
• Index date for considering the effect of different stages of pandemic, which was binned into different time intervals (March 2020 – June 2020, July 2020 – October 2020, November 2020 - February 2021, March 2021 – June 2021, July 2021 – November 2021).
• Medical utilizations measured by numbers of inpatient, outpatient, and emergency encounters in the baseline period (binned into 0 visit, 1 or 2 visits, 3 or 4 visits, 5+ visits for each encounter type).
• Coexisting conditions including comorbidities and medications based on a tailored list of the Elixhauser comorbidities. We defined the patient having a particular condition if he/she had at least two related records during the baseline period.

For identifying the negative controls for each patient in a particular subphenotype, we first required exact match for confounders of demographics, ADI, and index date to obtain an initial set, and then performed robust propensity score (PS) matching on other hypothetical confounders robust propensity score to rank the patients in the initial set and we finally picked the top 2.

The detailed description of this procedure was provided in Methods.

Blinding

This is an observational study and thus blinding is not possible.

Reporting for specific materials, systems and methods

We require information from authors about some types of materials, experimental systems and methods used in many studies. Here, indicate whether each material, system or method listed is relevant to your study. If you are not sure if a list item applies to your research, read the appropriate section before selecting a response.

Materials & experimental systems

n/a	Involved in the study	
☒	☐	Antibodies
☒	☐	Eukaryotic cell lines
☒	☐	Palaeontology and archaeology
☒	☐	Animals and other organisms
☐	☒	Clinical data
☒	☐	Dual use research of concern

Methods

n/a	Involved in the study	
☒	☐	ChIP-seq
☒	☐	Flow cytometry
☒	☐	MRI-based neuroimaging

Clinical data

Policy information about clinical studies
All manuscripts should comply with the ICMJE guidelines for publication of clinical research and a completed CONSORT checklist must be included with all submissions.

Clinical trial registration

Not a clinical trial

Study protocol

n/a, not a clinical trial

Data collection

Our study included 20,881 adult patients from the INSIGHT clinical research network (CRN) and 13,724 adult patients from the OneFlorida+ CRN who tested positive for SARS-CoV-2 on viral tests between March 01, 2020 and November 30, 2021

Outcomes

Newly incident diagnosis in the post-acute SARS-CoV-2 infection period (30-180 days after COVID confirmation)
